# Goodness-of-Fit Tests and Nonparametric Adaptive Estimation for Spike Train Analysis

**DOI:** 10.1186/2190-8567-4-3

**Published:** 2014-04-17

**Authors:** Patricia Reynaud-Bouret, Vincent Rivoirard, Franck Grammont, Christine Tuleau-Malot

**Affiliations:** 1 CNRS, LJAD, UMR 7351, Université Nice Sophia Antipolis, 06100, Nice, France; 2 CEREMADE UMR CNRS 7534, Université Paris Dauphine, Place du Maréchal De Lattre De Tassigny, 75775, Paris Cedex 16, France

## Abstract

When dealing with classical spike train analysis, the practitioner often performs goodness-of-fit tests to test whether the observed process is a Poisson process, for instance, or if it obeys another type of probabilistic model (Yana et al. in Biophys. J. 46(3):323–330, 1984; Brown et al. in Neural Comput. 14(2):325–346, 2002; Pouzat and Chaffiol in Technical report, http://arxiv.org/abs/arXiv:0909.2785, 2009). In doing so, there is a fundamental plug-in step, where the parameters of the supposed underlying model are estimated. The aim of this article is to show that plug-in has sometimes very undesirable effects. We propose a new method based on subsampling to deal with those plug-in issues in the case of the Kolmogorov–Smirnov test of uniformity. The method relies on the plug-in of good estimates of the underlying model that have to be consistent with a controlled rate of convergence. Some nonparametric estimates satisfying those constraints in the Poisson or in the Hawkes framework are highlighted. Moreover, they share adaptive properties that are useful from a practical point of view. We show the performance of those methods on simulated data. We also provide a complete analysis with these tools on single unit activity recorded on a monkey during a sensory-motor task.

Electronic Supplementary Material

The online version of this article (doi:10.1186/2190-8567-4-3) contains supplementary material.

## 1 Introduction

In neuroscience, the action potentials (spikes) are the main components for the real-time information processing in the brain. Moreover, it is possible to record in vivo several neurons and to have access to simultaneous spike trains. The duration of each spike is very small, about one millisecond. Moreover, the number and the position of each spike fluctuate from one trial to another trial. It is consequently quite natural to assimilate a spike to a random event. Therefore, in this article, we mathematically model spike trains as real-valued *point processes* that have been deeply described and studied for a long time in the literature (see [4] for a review) and often used in neuroscience (see, for instance, [2] and the references therein). However, except in very particular tests of independence (see, for instance, [5,6]), it is most of the time necessary to describe spike trains as realizations of particular stochastic processes. 

Most of the analyses start by answering a standard basic question. Is the process an homogeneous Poisson process or not? See, for instance, [7-9]. Indeed, for this simple model, extensively used in neuroscience, there is only one parameter to infer, namely the *firing rate*. The study of firing rates in neuroscience has lead to significative advances in the understanding of the coding of the direction of movements [10] for instance. But most of the time, spikes trains are more complex than homogeneous Poisson processes. Various studies have exhibited different kinds of correlations between some motor, sensory, or cognitive events in a behaving animal and a variation of the firing rate of specific neurons, before, during or after this event [11,12]. In particular, such data cannot be stationary. So, constraints on the previous model are relaxed and processes can be assumed to be *inhomogeneous Poisson processes*. In this setting, the firing rate is now time-dependent and is modeled by a function λ(⋅), which is the intensity of the inhomogeneous Poisson process (see [8,9]). Several studies have also established statistical evidence of dependence between the occurrences of the spikes of several neurons (see [5,6,13-15]) or even within a given neuron. In this case, standard homogeneous or inhomogeneous Poisson processes cannot be used and models based on *univariate or multivariate Hawkes processes* or variations of them are quite natural to capture dependence of spikes occurrences [16-21]. Hawkes processes, extensively described and discussed later on, generalize homogeneous Poisson processes by using functions quantifying interactions between spikes. These functions are called *interaction functions*. Such interaction functions are used in neuroscience to model excitation and inhibition phenomena [22]. 

Whatever the chosen model, this model has to be tested before any other inference based on this model. A plug-in step to infer unknown parameters is most of the time unavoidable to perform these tests. More precisely, for general models on point processes, the main ingredient consists in transforming the data so that the time changed process becomes a homogeneous Poisson process, fact which can be easily tested. However, the parameters of the transformation are usually unknown and are replaced by estimates. This plug-in trick has been widely popularized since [23]. It is widely used in neuroscience since [1] (see also the textbook of Tuckwell [24], [3], or [2]). The main goal of this article is to precisely show that the plug-in step may sometimes lead to undesirable effects and to propose the subsampling as a reasonable and quite universal solution. We focus here on the Kolmogorov–Smirnov (K.S.) test of uniformity. Indeed this K.S. test is usually considered as one of the three main tests on the first-order statistics that can be done to test the homogeneous Poisson hypothesis (see [1] and the references therein). More generally, the K.S. test (see [25] for its first use up to our knowledge) is one of the main omnibus tests [26], meaning that it is effective against a wide class of alternatives. However, it is known that a plug-in has to be taken with care for this test (see [27] for some brief discussion of this point). By using aggregated or cumulated tests, we propose 5 tests based on subsampling as goodness-of-fit tests, for which plug-in issues are solved. Note that, in neuroscience, plug-in problems have already been emphasized for other types of tests, namely the independence tests [22]. 

The second goal of this paper results from the first one: We have to develop statistical methods in the setting of point processes to estimate functions such as the intensity for the Poisson model or the interaction functions for the Hawkes model. Standard statistical procedures consist in assuming that these functions are parameterized by a few number of parameters, and in taking (for instance) the maximum likelihood estimator [28,29]. This approach is called *parametric*. For instance, assuming that a spike train is an homogeneous Poisson process, is equivalent to parameterizing the intensity by one parameter, namely the fixed constant firing rate. However, in neuroscience, except in the particular case of the homogeneous Poisson process, there is no a priori parametric shape for the functions to be estimated. These functions are most of the time unknown. Our second main contribution consists in proposing estimation procedures in a very flexible setting once the probabilistic model is fixed. So we consider the setting of *nonparametric* statistics, which is designed to estimate functions when no parametric model can be assumed. In particular, this nonparametric setting allows us to weaken assumptions considerably. The estimates proposed in this paper are based on kernel rules, wavelets expansions, or penalized criteria. Not only are they nonparametric, but they also share the following features: 

1. They are obtained by completely data-driven procedures that can be used even by neophytes in nonparametric statistics.

2. They achieve optimal convergence rates.

3. They do not assume light tails or any shape (exponential, unimodal, etc.) about the underlying function.

4. They adapt to the smoothness of the underlying function.

 Furthermore, the developed strategies considerably extend the procedures proposed by [7,30]. In particular, new data-driven kernel rules are introduced to estimate the intensity of inhomogeneous Poisson processes. We also derive a lasso-type estimate for recovering interaction functions of multivariate Hawkes processes when observing *n* trials. Some new interpretations of the estimate and connections with classical tools of the neuroscience literature such as joint peristimulus time histograms (JPSTH) and cross correlograms are also proposed. Theoretical results are established by using the *oracle* approach (see later).

The article is organized as follows. We first explain how subsampling can overcome the issues raised by plug-in for goodness-of-fit tests for the special case of the K.S. test. Then we extensively discuss adaptive nonparametric estimation and its advantages with respect to parametric estimation. This is illustrated on Poisson or Hawkes processes and a wide range of nonparametric methods are proposed. Finally, some simulations have been performed and real data sets coming from the recordings of a sensory-motor task (that can be found in [15], for instance) are analyzed thanks to these new methods. Most of the analysis has been performed with the software R. We refer to [7] for a complete list of its advantages. 

Let us introduce succinctly the main notions. More mathematical insight on the subject can also be found in [31]. For more-to-the-point definitions in link with neuroscience, and heuristic interpretations, we refer the interested reader to the very limpid article of Brown et al. [2] on the time-rescaling theorem. In the sequel, a point process *N* is a random countable set of points. For all measurable subset *A*, N(A) is the random variable giving the number of points of *N* in *A*. The associated point measure *dN* is defined as follows: for all measurable function *f*,

$$\int f(x)dN(x)=\sum_{T\in N}f(T).$$

To a finite point process *N* on the positive real line, one can associate the corresponding counting process ${\left(N_{t}\right)}_{t\geq 0}={\left(N([0,t])\right)}_{t\geq 0}$ and its compensator ${\left(\Lambda (t)\right)}_{t\geq 0}$ with respect to some given filtration (history). Most of the time, a conditional intensity λ(⋅) depending on the past history exists and in this case

$$\Lambda (t)=\int_{0}^{t}\lambda (u)du.$$

The function Λ(⋅) is therefore continuous nondecreasing. This is also the time-transformation on which the time-rescaling theorem is based [2]. In the sequel, $X_{p}\overset{P}{\underset{p\to \infty }{\to }}0$ means that the sequence $X_{p}$ converges in probability toward 0 when *p* tends to infinity; $X_{p}\overset{L}{\underset{p\to \infty }{\to }}X$ means that the distribution of $X_{p}$ tends to the one of *X* when *p* tends to infinity.

## 2 Goodness-of-Fit Tests: The Plug-in Drawback and Subsampling as a Possible Universal Solution

Once spike trains have been obtained and sorted, neurophysiologists often perform a very basic data analysis, which consists in testing several features such as stationarity for instance among other statistical inferences [7]. Following Ventura et al. [8], the first step of a “good practice” is usually to test whether the observed spike train is homogeneous Poisson or not. But it is usually admitted that real spike trains cannot be that simple and this hypothesis is most of the time rejected. To explain the rejection, the next step, still following [8], is to impute it to a lack of stationarity or to something more complex. It means that we have to test whether the process is an inhomogeneous Poisson process or not. For this purpose, one uses the time-rescaling theorem (see [32] but also [4,31]) under the hypothesis that the process is a Poisson process with deterministic intensity λ(⋅). Its associated compensator Λ(⋅) is in this case deterministic as well. The time-rescaling theorem, in its simplest version, states therefore that if *N* is a Poisson process with intensity λ(⋅), observed on $\left[0,T_{\max}\right]$, then N={X=Λ(T):T∈N} is an homogeneous Poisson process on $\left[0,\Lambda (T_{\max})\right]$ with intensity 1, fact which can be tested by practitioners. However, there is a misspecification in the method since λ(⋅) is unknown. The most popular and widely used method in neuroscience consists in plugging an estimate $\hat{\lambda }(\cdot )$ in [8]. As explained in the Introduction, we first illustrate the drawbacks of noncautious plug-ins for goodness-of-fit tests on the K.S. test, which has already been observed by [27]. We then propose a remedy to overcome these drawbacks based on subsampling. 

### 2.1 Elementary Situation for Illustration

Let us illustrate our purpose on a very basic situation. Assume that one observes $X_{1},\ldots ,X_{n}$*n* independent and identically distributed (i.i.d.) real variables with cumulative distribution function (c.d.f.) $u\to F(u)=P(X_{1}\leq u)$. Given $F_{0}$ a c.d.f., we can test whether the hypothesis $H_{0}$: “$F=F_{0}$” is true or not. To do so, let us first define $F_{n}$ the empirical distribution function associated with the $X_{i}$’s by

$$u\to F_{n}(u)=\frac{1}{n}\sum_{i=1}^{n}1_{\left\{X_{i}\leq u\right\}}.$$

If *n* is large enough, $F_{n}(u)$ is close to F(u) for any *u*. The K.S. test is therefore based on the statistic

(1) $${\mathrm{KS}}_{n}=\underset{u}{\sup}\left|F_{n}(u)-F_{0}(u)\right|.$$

Under $H_{0}$, if $F_{0}$ is continuous, the distribution of ${\mathrm{KS}}_{n}$ is known and it does not depend on $F_{0}$, so it can be tabulated [27]. For any α∈(0,1), let $k_{n,1-\alpha }$ be the 1−α quantile of this distribution. The classical (without plug-in) K.S. test consists in rejecting $H_{0}$ whenever ${\mathrm{KS}}_{n}>k_{n,1-\alpha }$ and this test is of exact level *α*. Note also that when *n* tends to ∞, the random variable $\sqrt{n}{\mathrm{KS}}_{n}$ tends in distribution to a tabulated distribution  (see [33]). As a consequence, if ${\tilde{k}}_{1-\alpha }$ is the 1−α quantile of , $\sqrt{n}k_{n,1-\alpha }$ tends to ${\tilde{k}}_{1-\alpha }$ and the approximation is valid as soon as n>45[34] (see also Durbin’s modification in [27]). 

Often, the c.d.f. $F_{0}$ is unknown since it depends on one or several unknown parameters and a natural idea consists in estimating it to use the previous procedure. This idea, extensively used in neuroscience, can lead to false results. For illustration, assume for example that we wish to test the hypothesis $H_{0}$ “the $X_{i}$’s are exponential with unknown parameter *λ*.” Note that this hypothesis is often tested on the interspike time intervals (ISI) [24] in order to test whether the observed spike process is an homogeneous Poisson process with unknown intensity *λ*. Following the scheme described previously, a natural procedure to test the exponentiality of the $X_{i}$’s could be the following: 

(i) Estimate *λ* by $\hat{\lambda }=1/\bar{X}$, where $\bar{X}$ is the empirical mean of the $X_{i}$’s: $\bar{X}=n^{-1}\sum_{i=1}^{n}X_{i}$.

(ii) Plug in the estimate $\hat{\lambda }$ and estimate $F_{0}$ by $u\to \hat{F}(u)=1-\exp(-\hat{\lambda }u)$.

(iii) Form the K.S. statistic (1) by replacing $F_{0}$ by $\hat{F}$. This leads to ${\mathrm{KS}}^{\left(1\right)}$.

(iv) Reject $H_{0}$ whenever ${\mathrm{KS}}^{\left(1\right)}>k_{n,1-\alpha }$.

**Fig. 1:**
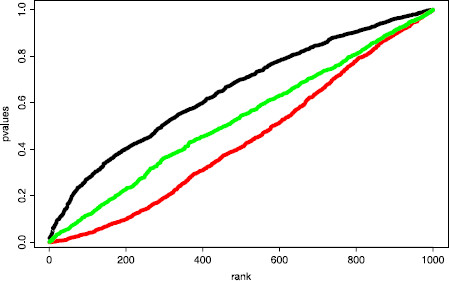
Repartition of the *p*-values in a K.S. test of exponentiality with plug-in. Graph of the *p*-values as a function of their rank. A n=40 i.i.d. sample of exponential variables with parameter λ=20 has been drawn 1000 times. Each time a *p*-value has been computed either by estimating the parameter *λ* and performing the K.S. test with exactly the same sample (*in black*), or by estimating the parameter *λ* on half of the sample and performing the K.S. test on the other half (*in red*), or by estimating the parameter *λ* on the whole sample and performing the K.S. test on a subsample of size $n^{2/3}$ (*in green*). Note that the estimated level (i.e., the number of *p*-values smaller than 0.05 divided by 1000) is in the first case of 0.009, of 0.12 in the second case, and of 0.039 in the third case. Those levels and curves are stable with respect to the sample size: Similar results are obtained for a larger sample size (n=200 and n=1000)

The *p*-values of this test are represented in Fig. 1. If the distribution of the test statistic was correctly predicted by the quantiles $k_{n,1-\alpha }$, then the repartition of the *p*-values should be close to the first diagonal of the graph (see [35]). Clearly, the curve is above the diagonal and the test is too conservative, which means that the test will accept $H_{0}$ more than required. The previous procedure fails in obtaining good results since, roughly speaking, the same data are used to estimate *λ* and to compute the test statistic. For very specific c.d.f., this problem can be overcome by computing the distribution of ${\mathrm{KS}}^{\left(1\right)}$ (see [27] for exponential and Gaussian cases). However, this is based on a trick that makes distributions, in those specific cases, independent of the unknown underlying parameter *λ*. Therefore, this solution cannot be adapted to complex situations such as the inhomogeneous Poisson process framework described above in the neuroscience field [8]. 

To be more careful and to avoid dependencies between $\hat{\lambda }$ and $F_{n}$, we could use the following “split into two parts” procedure where *n* is assumed to be even. 

(i) Estimate *λ* by $\tilde{\lambda }=1/\bar{\bar{X}}$, where $\bar{\bar{X}}$ is the empirical mean of the first half of the $X_{i}$’s: $\bar{\bar{X}}=2/n\sum_{i=1}^{n/2}X_{i}$.

(ii) Plug in the estimate $\tilde{\lambda }$ and estimate $F_{0}$ by $u\to \tilde{F}(u)=1-\exp(-\tilde{\lambda }u)$.

(iii) Form the K.S. statistic (1) by replacing $F_{0}$ by $\tilde{F}$, but also by replacing $F_{n}$ by the empirical cumulative distribution function only based on $X_{n/2+1},\ldots ,X_{n}$. This leads to ${\mathrm{KS}}^{\left(2\right)}$.

(iv) Reject $H_{0}$ whenever ${\mathrm{KS}}^{\left(2\right)}>k_{n/2,1-\alpha }$.

The *p*-values of this test are represented on Fig. 1. Surprisingly, the distribution of the *p*-values shows that the resulting test is not conservative enough. Indeed, the test will reject $H_{0}$ more than required and this procedure is even worse than the first strategy. Therefore, we turn toward a much more universal strategy, subsampling, thanks to the following result (see the Additional File 1 for the proof).

**Proposition 1***Let*$X_{1},\ldots ,X_{p}$*bepi*.*i*.*d*. *variables with c*.*d*.*f*. *Fassumed to be continuous*. *Let*$F_{p}$*be the associated empirical distribution*. *Assume that*$\hat{F}$*is a consistent estimate ofFsuch that*

(2) $$\sqrt{p}\underset{x}{\sup}\left|\hat{F}(x)-F(x)\right|\overset{P}{\underset{p\to \infty }{\to }}0.$$

Then

$$\sqrt{p}\underset{x}{\sup}\left|F_{p}(x)-\hat{F}(x)\right|\overset{L}{\underset{p\to \infty }{\to }}K.$$

Therefore, it remains to find $\hat{F}$ satisfying (2). In most of the parametric cases, and in particular in the exponential case, Assumption (2) does not hold if $\hat{F}$ is based on the same data as $F_{p}$. Assumption (2) may hold if *p* is much smaller than *n*, the whole sample size, as illustrated by the following strategy. 

Technical arguments of Additional File 1 prove that the previous test is of exact level *α* asymptotically. More importantly, in practice this conclusion remains true even for relatively small values of *n* as shown in Fig. 1 illustrated with n=40. Even if this test is not as powerful as the one described in [27], it has the main advantage to be almost universal. It can be adapted to most of parametric situations, since the use of subsampling makes the condition (2) quite easy to fulfill.

We want now to adapt this method to the more general scheme of goodness-of-fit tests for counting processes. From now on and whatever the situation, *p* will always correspond to the size of a subsample, i.e., a positive integer much smaller than *n* the total number of observations.

### 2.2 Aggregated Test of $H_{0}$: “The Observed Processes Are i.i.d. Poisson Processes”

To fix notation, we consider in the sequel that we observe *n* i.i.d. trials. Consequently, we have access to $N_{1},\ldots ,N_{n}$, *n* i.i.d. point processes observed on $\left[0,T_{\max}\right]$ representing the *n* i.i.d. spike trains of a fixed recorded neuron during $T_{\max}$ seconds.

It is not possible to assess on just one realization whether a point process is a (non necessarily homogeneous) Poisson process or not since the variations of the repartition of the points between different parts of one trial can either be due to nonstationarity or to more complex dependency structures that cannot be studied on just one run.

The first simple way to use the repetition of the trials is to use aggregation, which can be viewed as an empirical sum on the point processes. More precisely, the aggregated process over the processes $N_{1},\ldots ,N_{p}$ is defined by

$$N^{a,p}=\underset{i=1,\ldots ,p}{⋃}N_{i}\;\text{or equivalently}\;dN^{a,p}=\sum_{i=1}^{p}dN_{i}.$$

By classical properties of Poisson processes [4], if the processes are i.i.d. Poisson processes with compensator Λ(⋅), then $N^{a,p}$ is also a Poisson process with compensator pΛ(⋅). This implies that conditionally to the event $\left\{N^{a,p}([0,T_{\max}])=n_{\mathrm{tot}}\right\}$, the observed points of $N^{a,p}$ behave like an $n_{\mathrm{tot}}$ i.i.d. sample of c.d.f.

$$t\to F(t)=\frac{\Lambda (t)}{\Lambda (T_{\max})}.$$

Since *F* is unknown in our present situation, one has to estimate it, which leads to exactly the same plug-in problem as before. Fortunately, we are able to prove the following result.

**Proposition 2***Let*$N_{1},\ldots ,N_{p}$*bepi*.*i*.*d*. *Poisson processes with compensator*Λ(⋅), *assumed to be continuous*, *on*$\left[0,T_{\max}\right]$. *Let*$F_{N^{a,p}([0,T_{\max}])}$*be the associated empirical distribution*, *defined for anyxby*

(3) $$F_{N^{a,p}([0,T_{\max}])}(x)=\frac{1}{N^{a,p}([0,T_{\max}])}\sum_{T\in N^{a,p}}1_{\left\{T\leq x\right\}},$$

*where*$N^{a,p}$*is the aggregated Poisson process*. *Assume that*$\hat{F}(\cdot )$*is a consistent estimate of*$F(\cdot )=\Lambda (\cdot )/\Lambda (T_{\max})$*such that*

(4) $$\sqrt{N^{a,p}\left([0,T_{\max}]\right)}\underset{x\in [0,T_{\max}]}{\sup}\left|\hat{F}(x)-F(x)\right|\overset{P}{\underset{p\to \infty }{\to }}0.$$

Then

$$\sqrt{N^{a,p}\left([0,T_{\max}]\right)}\underset{x\in [0,T_{\max}]}{\sup}|F_{N^{a,p}([0,T_{\max}])}(x)-\hat{F}(x)|\overset{L}{\underset{p\to \infty }{\to }}K.$$

Once again, subsampling (i.e., choosing *p* much smaller than *n*) gives us estimates $\hat{F}$ satisfying (4). Two different approaches lead to two distinct tests. First, let us use the empirical c.d.f. on the whole sample. 

In Additional File 1, we prove that this test is of exact asymptotic level *α*, as soon as the compensator Λ(⋅) is continuous and this even if λ(⋅) does not exist. However its practical performance are poor (see later). A slightly more useful test can be obtained by using smoother and more elaborate estimates $\hat{F}$ satisfying (4). We obtain the following testing procedure. 

In Additional File 1, we prove that the previous test is of asymptotic level *α*. Note that Condition (5) can be demanding and rejection can be due to nonfulfillment of this condition. For instance, estimates $\hat{\lambda }$ based on parametric estimates on a prescribed parametric model (such as maximum likelihood estimates for instance, see [8]) fulfill (5) if the prescribed model is true, but cannot fulfill this condition if the prescribed parametric model is not true. Hence, using parametric estimates in this setting lead to test both $H_{0}$ and “the prescribed parametric model is correct,” which is not satisfying. Therefore, it is natural to make no parametric assumption on the underlying model for λ(⋅) and to try to fulfill (5) by using nonparametric estimation.

Finally, as already observed by [8], aggregation can dilute the dependencies between the points. Therefore, Tests 2 and 3 cannot be really powerful as we will see later.

### 2.3 Cumulated Goodness-of-Fit Tests

**Fig. 2:**
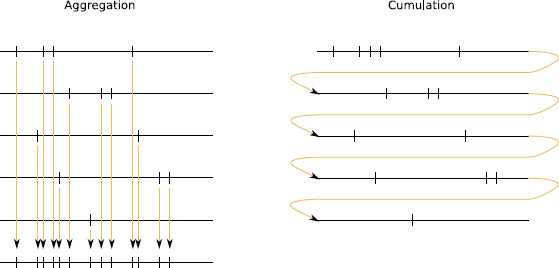
Aggregation versus cumulation. Description of the way the points are gathered together for aggregation or for cumulation. *On the left-hand side*, *the first five lines* correspond to a trial, *the sixth line* being the aggregated process. *On the right-hand side*, *the same five lines* are put together to form the cumulated process

Another way to use the repetition of the trials, is to cumulate the *p* processes instead of aggregating them. This difference is made more explicit in Fig. 2. When processes are aggregated, points of very different trials can be very close, which can dilute dependencies between occurrences. This cannot occur for cumulated processes. With this method, we can also test models that are more general than Poisson processes. 

Those general models are usually described through their conditional intensity λ(⋅), which represents the probability of occurrence of a point at time *t* given the past before *t*. So, defining a model through its conditional intensity is the easiest way to model the dependence between points. For instance, when we assume the conditional intensity *λ* to be a deterministic function *f*, we are assuming independence with respect to the past. This is equivalent to assuming that the process is an inhomogeneous Poisson process with intensity λ=f. Therefore, testing $H_{0}$: “the observed processes are i.i.d. with conditional intensity λ=f and unknown deterministic function *f*” is equivalent to testing $H_{0}$: “the observed processes are i.i.d. inhomogeneous Poisson processes.”

More generally, we wish to test a nonparametric hypothesis on the conditional intensity. An example, more developed in the next section, is the multivariate Hawkes process, which models the dependence between the spikes of different neurons via several interaction functions, for which we do not want to give a parametric form. Let us give just a simple expression of this process to illustrate our set-up, with only one process. The classical self-exciting Hawkes process has conditional intensity given by

(6) $$\lambda (t)=\lambda_{f}(t)=\mu +\int_{-\infty }^{t-}h(t-u)dN(u),$$

where *μ* is a positive real parameter and *h* a non negative integrable function with support in $R_{+}^{*}$ and with f=(μ,h). For instance, if the function *h* is supported by the interval (0,2], then the probability of occurrence at time *t* randomly depends on the occurrences of the process on [t−2,t). Testing whether the process is a classical self-exciting Hawkes or not can be rephrased as testing whether the process has conditional intensity given by the form $\lambda_{f}$ defined in (6), with unknown *f*. Other famous examples in biomedical areas such as the multiplicative Aalen intensity or the Cox model can be found in [29]. 

As in the previous subsection, we use the time-rescaling theorem but in a deeper way. Remember that the general time-rescaling theorem [2] states that for any point process *N* on $\left[0,T_{\max}\right]$ with compensator Λ(⋅), the point process N={X=Λ(T):T∈N} is a Poisson process with intensity 1 on $\left[0,\Lambda (T_{\max})\right]$. Therefore, it is more interesting to cumulate the processes after time-rescaling than in the usual time space $\left[0,T_{\max}\right]$. For general conditional intensity models, Λ(⋅) is random. Therefore the state space $\left[0,\Lambda (T_{\max})\right]$ is also random in general. Moreover, when we are dealing with *p* i.i.d. processes $N_{1},\ldots ,N_{p}$, each $N_{i}$ has a different compensator $\Lambda_{i}(\cdot )$ which depends on the history of the *i*th trial. So except in the Poisson case where Λ(⋅) is deterministic, we do not apply the same transformation to all the points. We finally have to deal with *p* processes $N_{i}=\{X=\Lambda_{i}(T):T\in N_{i}\}$ that are Poisson processes of intensity 1, and whose occurrences lie in $\left[0,\Lambda_{i}(T_{\max})\right]$. Even if the $\Lambda_{i}(T_{\max})$ are i.i.d., they are not equal in general.

This leads to two main remarks. First, it is not possible to aggregate in general the time-transformed processes since we would aggregate processes with different lengths (see Fig. 2). Therefore, Tests 2 and 3 cannot be transferred to the most general case straightforwardly. However, one can cumulate those processes as done in Fig. 2 and this even if the intervals have different lengths. The resulting process $N^{c,p}$ is therefore a Poisson process with intensity 1 on the random interval $I=[0,\sum_{i=1}^{p}\Lambda_{i}(T_{\max})]$ (see also Additional File 1 for a more precise formula and a proof of this statement). The second remark consists in noting that $\sum_{i=1}^{p}\Lambda_{i}(T_{\max})$ being a random quantity, it is not true in general that conditionally to the total number of points in ℐ, the points of $N^{c,p}$ behave like an i.i.d. uniform sample, and in the sequel we shall need to restrict ourselves to an interval of the form [0,pθ] with a deterministic bound *pθ*, which is with high probability, smaller than $\sum_{i=1}^{p}\Lambda_{i}(T_{\max})$.

Besides we have to deal with estimation of unknown transformations $\Lambda_{i}(\cdot )$. For this purpose, we introduce estimates of the type $t\to {\hat{\Lambda }}_{i}(t)=\int_{0}^{t}{\hat{\lambda }}_{i}(u)du$, where ${\hat{\lambda }}_{i}(\cdot )$ estimates $\lambda_{i}(\cdot )$, the conditional intensity of the *i*th process $N_{i}$. We obtain a cumulate process ${\hat{N}}^{c,p}$ built from the ${\hat{\Lambda }}_{i}(\cdot )$’s. We have the following equivalent to Proposition 2.

**Theorem 1***Let*$N_{1},\ldots ,N_{p}$*bepi*.*i*.*d*. *processes with respective conditional intensity*$\lambda_{i}(\cdot )$. *Assume that there exist nonnegative estimates*${\hat{\lambda }}_{i}(\cdot )$*of*$\lambda_{i}(\cdot )$*such that*

(7) $$p^{-1/2}\left(\sum_{i=1}^{p}\int_{0}^{T_{\max}}|{\hat{\lambda }}_{i}(u)-\lambda_{i}(u)|du\right)\overset{P}{\underset{p\to \infty }{\to }}0.$$

*Then*, *for all*θ>0*such that*$E(\Lambda_{1}(T_{\max}))>\theta$,

$$\sqrt{{\hat{N}}^{c,p}\left([0,p\theta ]\right)}\underset{u\in [0,1]}{\sup}|\frac{1}{{\hat{N}}^{c,p}([0,p\theta ])}\sum_{X\in {\hat{N}}^{c,p},X\leq p\theta }1_{\left\{X/(p\theta )\leq u\right\}}-u|\overset{L}{\underset{p\to \infty }{\to }}K.$$

It is now easy to turn this result into an operational test, using subsampling. 

In Additional File 1, we prove that the previous test is of exact asymptotic level *α* as soon as $E(\Lambda_{i}(T_{\max}))>\theta$. There exists a simpler form of this test when dealing with Poisson processes since in this case compensators do not depend on *i*. 

This test, as a special case of Test 4, is also of exact asymptotic level *α* as soon as $\Lambda (T_{\max})>\theta$. Tests 4 and 5 are more powerful to detect dependencies or to reject the Poisson assumption than Tests 2 or 3, as we will see later.

As for Test 3, and for exactly the same reasons, we want to find nonparametric estimates satisfying (8) or (9). We provide in the next section powerful tools to deal with this problem and theoretical guarantees of performance of these estimates.

## 3 Nonparametric and Adaptive Estimation

### 3.1 Why Is Adaptive Estimation Useful?

Nonparametric estimation, and in particular nonparametric estimation of Poisson process intensity, is at the root of most of the data analyses performed on spike trains. Indeed, peristimulus time histograms (PSTH) [36] are usually the first graphical representations of an experiment. Those histograms have usually a fixed length for each interval (typically 10 ms) and are quite noisy from a statistical point of view (see, for instance, the representations of [8]). Therefore, there have been several attempts to provide smoother estimates, either by kernel estimates (see, for instance, [30]) or by projection on an orthonormal basis (see, for instance, [7] for the use of splines). These methods provide a first illustration of the data with as few assumptions as possible on the underlying “true” firing rate. They are originally not linked at all to any statistical or probabilistic models and constitute descriptive statistics. In particular, no parametric assumption on the underlying intensity is made at this step, the parametric model and its associated maximum likelihood estimator (MLE) being given in a second time once the shape of the curve is guessed [8]. Because of this lack of parametric assumption, those estimates seem to be the best candidates at first glance for the estimate $\hat{\lambda }$ that needs to be plugged in Tests 3 or 5.

However, the problem of the convergence rate remains. In all these methods, there is a tuning parameter that needs to be chosen: it is the length of the interval for histograms, the bandwidth in kernel rules or the number of coefficients in the orthonormal expansion. The problem of the choice of this parameter has first been tackled very roughly in the neuroscience literature by choosing a fixed value. On the real data presented here or on the ones in [8], it was usually considered that a bandwidth of 50 or 100 ms was a good choice. However, such a very rough choice cannot guarantee a convergence rate when *n* goes to ∞. Indeed let us look more closely at the kernel estimate.

For the i.i.d. observed point processes $N_{1},\ldots ,N_{n}$, the kernel estimate with kernel *K* and bandwidth *h* is given by

(10) $${\hat{\lambda }}_{n}^{K_{h}}(x)=\frac{1}{n}\sum_{i=1}^{n}\int K_{h}(x-u)dN_{i}(u)=\frac{1}{n}\int K_{h}(x-u)dN^{a,n}(u),$$

where $N^{a,n}$ is the corresponding aggregated process and where $K_{h}(u)=(1/h)K(u/h)$. If we assume that the observed processes are inhomogeneous Poisson processes with intensity *λ*, $N^{a,n}$ is also an inhomogeneous Poisson process with intensity *nλ* and, therefore,

$$E\left[{\hat{\lambda }}_{n}^{K_{h}}(x)\right]=(K_{h}⋆\lambda )(x),\;\forall x\in R,$$

where ⋆ denotes the convolution product. So, the expectation of ${\hat{\lambda }}_{n}^{K_{h}}$ constitutes a regularized approximation of λ(⋅). To measure the performance of ${\hat{\lambda }}_{n}^{K_{h}}$, we compute its $L_{2}$-risk (see further details in Additional File 2):

(11) $$E{∥{\hat{\lambda }}_{n}^{K_{h}}-\lambda ∥}_{2}^{2}={∥K_{h}⋆\lambda -\lambda ∥}_{2}^{2}+\frac{{∥\lambda ∥}_{1}}{nh}{∥K∥}_{2}^{2},$$

which is classically interpreted as a bias-variance decomposition. Therefore, if *h* is fixed, the variance term goes to 0 whereas the bias remains fixed so that the $L_{2}$-risk of the estimate does not go to 0. Consequently, a fixed choice for the bandwidth is not convenient and it is essential to choose h=h(n) tending to 0 with *n*. The dependence of *h* with respect to *n* is a problem that has been extensively studied in the density framework, a setting close to the present one since conditionally to the total number of points, the observed points of $N^{a,n}$ behave like an i.i.d. sample of density $\lambda (\cdot )/\Lambda (T_{\max})$. We refer the reader to [37] for a review. The main conclusion of such a study is that if *λ* is regular and if the regularity is known then we are able to choose h(n) such that the $L_{2}$-risk tends to 0 at a known rate of convergence depending on the regularity. Furthermore, the larger the regularity, the faster the rate. Typically, if the *r*th derivative of *λ* is bounded in the $L_{2}$ sense, then it is possible to choose *K* and^a^$h(n)≍n^{-1/(2r+1)}$ such that the $L_{2}$-risk behaves as

$$E{∥{\hat{\lambda }}_{n}^{K_{h}}-\lambda ∥}_{2}^{2}≍n^{-2r/(2r+1)}.$$

In this setting, this choice can be applied to Tests 3 and 5, since the Markov inequality implies that (5) or (9) are satisfied with $p(n)=n^{\delta }$ and δ<(2r)/(2r+1). The choice r=1 gives δ<2/3 and r=2 gives δ<4/5.

Of course, in practice the choice of the bandwidth is capital. Since the smoothness of *λ* is unknown, the practitioner cannot use the previous choice. Furthermore, guessing the order of magnitude of h(n) is not enough to achieve good performance since the leading constant plays an essential role. Hence, the theoretical considerations developed before do not solve the practical problem. Several directions have been proposed to overcome this problem. One of the most famous ones consists in using leave-one-out or other cross-validation methods [30,38]: among a finite family of fixed bandwidths, such methods choose the best one in an asymptotic setting. However, to our knowledge, nothing can be said when the family of bandwidths is not fixed and some bandwidths tend to 0 with *n*. It is not clear at all that the resulting estimate achieves a prescribed rate and, therefore, it cannot be used for the proposed tests in particular. Other methods based on the rule of the thumb (and variations of it) have been proposed in the density or the Poisson setting [8,39], and in this case the resulting bandwidth is of the form $h(n)=Cn^{-1/5}$ for various possible choices of the constant *C*. Generally, those choices lead to poor results as noted by [8] (see also our the simulation study). 

Adaptive estimation [37] aims at tuning in a data-driven way the unknown parameters of those methods (kernels, histograms, etc.) such that the resulting estimate has good practical performance and a guaranteed convergence rate. The adaptive estimates are usually mathematically proved to achieve the best possible rate of convergence and this even if the regularity is unknown. Moreover, they do not depend on any restrictive assumption such as, for instance, some parametric assumption. The only assumption lies in the underlying probabilistic model (for instance, one assumes that the processes are inhomogeneous Poisson processes). Their reconstructions are therefore much more trustworthy than other methods for which those extra assumptions may not be fulfilled. As a conclusion, adaptive estimates constitute ideal candidates to be plugged in Tests 3, 4, or 5. 

The main aim of next subsections is therefore to present adaptive estimates in the Poisson or in the Hawkes model that will have these good properties.

### 3.2 Adaptive Estimation for Poisson Processes

#### 3.2.1 Kernel Estimates

As mentioned previously, the Poisson setting is very close to the density setting. In the density setting, the main adaptive method for choosing a bandwidth is the Lepski method, which has been recently updated to the multidimensional framework and to deal with the problem of choosing the leading constant of the bandwidth. Due to Goldenshluger and Lepski [40], it is referred in the sequel as the GL method. We propose here to adapt this method to the Poisson setting in the following way and to prove its adaptive properties. 

We consider a set of bandwidths ℋ and their corresponding kernel estimates ${\hat{\lambda }}_{n}^{K_{h}}$. The bias-variance decomposition shows that the parameter $\bar{h}$ which minimizes the right-hand side of (11) with respect to h∈H is the best possible choice. It is called the oracle bandwidth: since it depends on λ(⋅), it cannot be used in practice. To propose a data-driven choice of the bandwidth by a GL method, we define for any $h,h^{\prime }\in H$:

$${\hat{\lambda }}_{n}^{h,h^{\prime }}(x):=\frac{1}{n}\sum_{T\in N^{a,n}}(K_{h}⋆K_{h^{\prime }})(x-T)=\left(K_{h}⋆{\hat{\lambda }}_{n}^{K_{h^{\prime }}}\right)(x),\;\forall x\in R,$$

then for η>0, we set

$$A(h):=\underset{h^{\prime }\in H}{\sup}{\left\{{∥{\hat{\lambda }}_{n}^{h,h^{\prime }}-{\hat{\lambda }}_{n}^{K_{h^{\prime }}}∥}_{2}-\frac{(1+\eta )(1+{∥K∥}_{1}){∥K∥}_{2}\sqrt{N^{a,n}([0,T_{\max}])}}{n\sqrt{h^{\prime }}}\right\}}_{+}.$$

The Additional File 2 shows that A(h) constitutes a good estimate of the bias term (see (11)). Finally, we select the data-driven bandwidth as follows:

(12) $$\hat{h}:=\arg\min_{h\in H}\left\{A(h)+\frac{(1+\eta )(1+{∥K∥}_{1}){∥K∥}_{2}\sqrt{N^{a,n}([0,T_{\max}])}}{n\sqrt{h}}\right\},$$

which allows us to estimate λ(⋅) by using

(13) $${\hat{\lambda }}_{n}^{\mathrm{GL}}:={\hat{\lambda }}_{n}^{K_{\hat{h}}}.$$

Note that in (12), ${∥K∥}_{2}^{2}N^{a,n}([0,T_{\max}])/(n^{2}h)$ is an unbiased estimate of the variance term in (11) and therefore the previous criterion mimics the bias-variance decomposition of the risk of ${\hat{\lambda }}_{n}^{K_{h}}$ up to some multiplicative constant. Once *K*, ℋ and *η* are chosen, we obtain a turnkey procedure. The following theoretical result justifies our procedure.

**Theorem 2***If*$H\subset \{D^{-1}:D=1,\ldots ,D_{\max}\}$*with*$D_{\max}=\delta n$*for some*δ>0, *and if*${∥\lambda ∥}_{\infty }<\infty$, *then*,

$$E{∥{\hat{\lambda }}_{n}^{\mathrm{GL}}-\lambda ∥}_{2}^{2}\leq C_{1}\underset{h\in H}{\inf}\left\{{∥K_{h}⋆\lambda -\lambda ∥}_{2}^{2}+\frac{{∥\lambda ∥}_{1}}{nh}{∥K∥}_{2}^{2}\right\}+C_{2}n^{-1},$$

*where*$C_{1}$*is a constant depending on*${∥K∥}_{1}$*andηand*$C_{2}$*is a constant depending onδ*, *η*, ${∥K∥}_{2}$, ${∥K∥}_{1}$, ${∥\lambda ∥}_{1}$, *and*${∥\lambda ∥}_{\infty }$.

Theorem 2 combined with (11) shows that our procedure mimics the performance of the oracle up to the constant $C_{1}$ and up to the term $C_{2}n^{-1}$, which is negligible when *n* goes to +∞. It is classically called an oracle inequality, which is the main property of adaptive estimates. In particular, one can take the family $H=\{1,\ldots ,{\left\lfloor \delta n\right\rfloor }^{-1}\}$, which grows with *n* and it is possible to select a bandwidth tending to 0 with *n*. If the *r*th derivative of λ(⋅) is bounded in $L_{2}$, then the choice $h(n)≍n^{-1/(2r+1)}$ is in the family ℋ and the oracle inequality gives straightforwardly that

$$E{∥{\hat{\lambda }}_{n}^{\mathrm{GL}}-\lambda ∥}_{2}^{2}≍n^{-2r/(2r+1)},$$

which is the optimal rate of convergence over such spaces. This rate is achieved, even if we do not know in advance the regularity *r* of *λ*, which is from a theoretical point of view the main improvement with respect to the theory described in the previous subsection.

If *K* is the Gaussian kernel, then ${∥K∥}_{1}=1$ and ${∥K∥}_{2}=2^{-1/2}\pi^{-1/4}$. Moreover, $K_{h}⋆K_{h^{\prime }}=K_{\sqrt{h^{2}+{h^{\prime }}^{2}}}$ and a straightforward computation shows that explicit formula for ${∥{\hat{\lambda }}_{n}^{h,h^{\prime }}-{\hat{\lambda }}_{n}^{K_{h^{\prime }}}∥}_{2}$ are also available. It is consequently very easy to implement the method, the computational cost being almost of the same order as cross-validation. We will see in the simulation study that this practical choice is also quite robust.

#### 3.2.2 Histograms

In the Poisson set-up, there are several ways to select data-driven partitions that lead to adaptive histogram estimates. For instance, one can use model selection as in [41]. Model selection can either select a regular partition or an irregular partition on a grid. When regular partitions are considered, the resulting estimator satisfies an oracle inequality similar to the oracle inequality established in Theorem 2 for the kernel rule combined with the GL method. Indeed the bin for the histograms plays exactly the same role as the kernel bandwidth. Therefore, it leads to similar theoretical performance, except that the histograms cannot become smooth enough to guarantee an optimal convergence rate for regular intensities (namely r>1). Therefore, the choice of regular partitions is probably not the best one and one may prefer the GL method. The data-driven choice of the partition becomes much more interesting when the partition is not forced to be regular. Indeed irregular partitions can capture a fast increase of the firing rate followed very quickly by a fast decrease at some particular moment of the experiment, without leading to too noisy estimates as the classical PSTH, since over smoother periods, the length of the interval can be much larger. However, the method of [41] is too time consuming to be really considered in practice. Another possible direction is the context of Markov modulated Poisson processes [42], where the algorithms are also quite time consuming without ensuring any adaptive property in terms of convergence rate (despite some possible interpretation with respect to hidden Markov processes). 

However, and as already noticed in [41], it is possible in certain cases to interpret a model selection estimate as a thresholding rule. We hereafter illustrate in a simpler case, the method developed in [43]: If $\lambda (\cdot )\in L_{2}$, we can decompose it on the Haar basis,

$$\lambda =\sum_{j=-1}^{+\infty }\sum_{k\in Z}\beta_{j,k}\psi_{j,k},$$

where $\psi_{-1,k}(\cdot )=\phi (\cdot -k)$ with $\phi =1_{\left[0,1\right)}$ the Haar father wavelet and where $\psi_{j,k}(\cdot )=2^{j/2}\psi (2^{j}(\cdot -k))$ for j≥0 with $\psi =1_{\left[0,1/2\right)}-1_{\left[1/2,1\right)}$ the Haar mother wavelet. The $\beta_{j,k}$’s are the unknown coefficients of λ(⋅) and are given by

$$\forall j\geq -1,k\in Z,\;\beta_{j,k}=\int \psi_{j,k}(x)\lambda (x)dx.$$

These coefficients can therefore be unbiasedly and consistently estimated by

$$\forall j\geq -1,k\in Z,\;{\hat{\beta }}_{j,k}=\frac{1}{n}\int \psi_{j,k}(x)dN^{a,n}(x).$$

Given a fixed finite subset of indices *m*, we obtain an easily computable estimate of λ(⋅):

$${\hat{\lambda }}_{n}^{m}=\sum_{(j,k)\in m}{\hat{\beta }}_{j,k}\psi_{j,k}.$$

Since the Haar basis is piecewise constant, the previous estimate is also piecewise constant on a certain partition  depending on *m*. A data-driven choice of *m* therefore leads to a data-driven choice of the partition that can be irregular. Let us fix an arbitrary highest level of resolution $j_{0}$ such that $2^{j_{0}}\leq n<2^{j_{0}+1}$ and let us consider the $L_{2}$-risk of ${\hat{\lambda }}_{n}^{m}$ such that if (j,k)∈m then $j\leq j_{0}$. The bias-variance decomposition of ${\hat{\lambda }}_{n}^{m}$ can be written as follows:

(14) $$\begin{array}{rcl} E\left[{∥{\hat{\lambda }}_{n}^{m}-\lambda ∥}^{2}\right] & = & \sum_{(j,k)\notin m}\beta_{j,k}^{2}+\sum_{(j,k)\in m}\mathrm{Var}({\hat{\beta }}_{j,k})\\ & = & \sum_{j>j_{0}}\sum_{k}\beta_{j,k}^{2}+\sum_{j\leq j_{0}}\sum_{k}\left[\beta_{j,k}^{2}1_{(j,k)\notin m}+v_{j,k}1_{(j,k)\in m}\right], \end{array}$$

where

$$v_{j,k}:=\mathrm{Var}({\hat{\beta }}_{j,k})=\frac{1}{n}\int \psi_{j,k}^{2}(x)\lambda (x)dx.$$

Hence, the best subset *m* is the set of indices (j,k) such that $\beta_{j,k}>\sqrt{v_{j,k}}$. This is the oracle choice. A possible data-driven way to choose the indices (j,k) is to choose the indices such that ${\hat{\beta }}_{j,k}$ are larger than a certain threshold $\eta_{jk}$ depending on an estimate of the variance $v_{j,k}$. The choice advertised in practice in [43] is

(15) $$\eta_{j,k}=\sqrt{2\gamma \ln(n){\hat{v}}_{j,k}}+\frac{\gamma \ln(n)2^{j/2}}{3n}\;\text{where }{\hat{v}}_{j,k}=\frac{1}{n^{2}}\int \psi_{j,k}^{2}(x)dN^{a,n}(x).$$

Then we obtain the following thresholding estimator:

(16) $${\hat{\lambda }}_{n}^{\mathrm{Th}}=\sum_{j=-1}^{j_{0}}\sum_{k}{\hat{\beta }}_{j,k}1_{\left\{|{\hat{\beta }}_{j,k}|>\eta_{j,k}\right\}}\psi_{j,k}.$$

In [43], it has been proved that a slight modification of this estimate satisfies an oracle inequality in the same spirit as Theorem 2. It also generalizes this estimate by considering general biorthogonal bases instead of the Haar basis, leading to smooth estimates (see [43,44]). In this case, for a slight modification of the threshold, the resulting estimate has the same convergence rates as the kernel estimate combined with the GL method, up to some logarithmic term, as soon γ>1. The choice γ<1 has been shown to lead to bad convergence rates and the choice γ=1 has been shown to work well on extensive simulations in both [43,44]. This method is easily implementable leading to very fast algorithms that are in particular faster than algorithms based on the GL method. 

#### 3.2.3 More Sophisticated Procedures

Thresholding rules and irregular partitions overcome a drawback of kernel estimates that suffer from a lack of spatial adaptivity on the time axis. Several attempts have been proposed to build more local choices of the bandwidth (see [30] for instance), but to our knowledge no mathematical proof of this spatial adaptation has been established, whereas histograms and in particular the previous Haar thresholding estimator can adapt the length of the bin to the heterogeneity of the data. But the resulting estimator is not smooth at all. As explained, we can consider a smoother wavelet basis, but this extension does not completely address the issue. 

The best alternative, to our knowledge, when the support of λ(⋅) is known and bounded (here $\left[0,T_{\max}\right]$) and when λ(⋅) does not vanish for a significant period of time, is due to Willett and Nowak [45]. Their method is quite intricate to describe. Informally, a penalized log-likelihood criterion is used to select a piecewise polynomial. Both the partition and the degree of each polynomial on each interval of the partition are free (on a very refined grid of resolution). Willett and Nowak have proved that such an estimator achieves optimal rates of convergence for various classes of regularity and in an adaptive way. From a practical point of view, a dyadic tree algorithm is used. Its complexity is much smaller than a full model selection method on the same piecewise polynomial family of models. It is a bit more complex than a thresholding algorithm, but there exist a program (FreeDegree) in Matlab interfaced with C which makes its use in practice quite easy. For a more complete description of the method, we refer to [45]. Note that in practice because of its adaptive properties, this estimator is able to be piecewise constant when the true intensity is piecewise constant but also very smooth (with high degree for the polynomials) when the underlying intensity is smooth and when the number of points is sufficient. It is also able to be spatially adaptive, the underlying data-driven partition being irregular. In the sequel, we denote this method ${\hat{\lambda }}_{n}^{\mathrm{WN}}$.

### 3.3 Adaptive Estimation for Hawkes Processes

If inhomogeneous Poisson processes can model nonstationary data, they are not appropriate to model dependencies between points. However, several studies have established potential dependence of spike occurrences for different neurons. This has been detected via descriptive statistics, via independence tests for a given fixed model or via model-free independence tests based on permutations (also called trials-shuffling) [5,6,13,15,22,46]. 

One simple model of dependency is the multivariate Hawkes process, which is the point process equivalent to the auto-regressive model. It has first been introduced by Hawkes [47], as a self-exciting point process, that is useful in particular in seismology (see, for instance, [23]). It has also been used to model positions of motifs along the DNA molecule [48,49]. In neuroscience, it explicitly appears in the 1980s with [19] and is close in spirit to [50,51], with the additional advantage of modeling potential feed-back between the neurons. 

The multivariate Hawkes process (see, for instance, [52] or [4]) models the instantaneous firing rates of *M* different neurons, with spike trains $N^{\left(1\right)},\ldots ,N^{\left(M\right)}$, where the conditional intensity of the *m*th point process is defined for any t≥0 by

(17) $$\begin{array}{rcl} \lambda^{\left(m\right)}(t) & = & {\left(\nu^{\left(m\right)}+\sum_{\ell =1}^{M}\int_{-\infty }^{t-}h_{\ell }^{\left(m\right)}(t-u)dN^{\left(\ell \right)}(u)\right)}_{+}\\ & = & {\left(\nu^{\left(m\right)}+\sum_{\ell =1}^{M}\sum_{T_{\ell }\in N^{\left(\ell \right)},T_{\ell }<t}h_{\ell }^{\left(m\right)}(t-T_{\ell })\right)}_{+}. \end{array}$$

In (17), the $\nu^{\left(m\right)}$’s are positive parameters representing the spontaneous firing rates and the $h_{\ell }^{\left(m\right)}$’s are the interaction functions and have support included into $R_{+}^{*}$. More precisely, before the first occurrence of the multivariate process, the $N^{\left(m\right)}$’s behave like homogeneous Poisson processes with constant intensities $\nu^{\left(m\right)}$. The first occurrence (and the next ones) affects all the processes by increasing or decreasing the conditional intensity via the interaction functions $h_{\ell }^{\left(m\right)}$’s. For instance, if $h_{\ell }^{\left(m\right)}$ takes large positive values in the neighborhood of the delay *d* and is null elsewhere, then after the delay *d* of one occurrence of $N^{\left(\ell \right)}$, the probability to have a new occurrence of $N^{\left(m\right)}$ will significantly increase: The process $N^{\left(\ell \right)}$ excites the process $N^{\left(m\right)}$. On the contrary, if $h_{\ell }^{\left(m\right)}$ is negative around *d*, then after the delay *d* of one occurrence of $N^{\left(\ell \right)}$, the probability to have a new occurrence of $N^{\left(m\right)}$ will significantly decrease: The process $N^{\left(\ell \right)}$ inhibits the process $N^{\left(m\right)}$. Note in particular that the functions $h_{m}^{\left(m\right)}$’s model self-interactions.

The Hawkes process as described above cannot really model nonstationary data. Indeed, when *t* grows (and under conditions on the interaction functions), the process converges quite quickly toward an equilibrium, which is stationary (see, for instance, [52,53], and the references therein). If these conditions are not satisfied, the number of points in the process grows too fast to be a realistic model for spike trains anyway. Hence, Hawkes processes as defined in (17) cannot model nonstationary data, but can model dependent data.

Therefore, we fix an interval $\left[T_{1},T_{2}]\subset [0,T_{\max}\right]$, typically an interval where all the estimated mean firing rates seem constant. The aim is to estimate on this interval

$$f^{*}=\left({\left(\nu^{\left(m\right)}\right)}_{m=1,\ldots ,M},{\left(h_{\ell }^{\left(m\right)}\right)}_{\ell ,m=1,\ldots ,M}\right),$$

where it is assumed that the interaction functions are bounded with support in [0,A] with $T_{1}>A$.

Inference for Hawkes models based on the likelihood can be found in the literature, in particular, for parametric models [23,49]. However, in neuroscience, for flexibility, the used parametric models are based on a large number of parameters. Therefore, they require several thousand spikes per neuron to be observed in a stationary way to achieve good estimation [19]. Classical model selection based on AIC and BIC criteria has also been used to select the number of knots for the spline estimate [21,48,54]. However, these criteria do not adapt well to irregular functions. This is the reason why alternative nonparametric adaptive inference has recently been developed in such models. The univariate case (M=1) has been studied in [55], where rates of convergence depending on the underlying regularity of the self-interaction function have been derived. We can also mention the alternatives proposed in [20,56] but no theoretical validation is provided in those works. 

A multivariate approach, valid for very general counting processes including Hawkes processes and based on $\ell_{1}$ penalties, has been recently developed in [57]. Based on minimization of convex criteria, its computational cost is more reasonable than procedures proposed in [55] and it is also proved to satisfy oracle inequalities. We shall detail this method in the case of Hawkes processes and with piecewise constant estimates of the underlying interaction functions. 

In the next section that can be skipped at first reading, we describe the method in a technical way. Then we give heuristic arguments to understand more deeply the presented method (see also [58] for a quicker view on this estimate). In particular, the method does not rely on the likelihood, but on a least-square contrast, which can be reinterpreted in terms of JPSTH [59]. 

#### 3.3.1 Intensity Candidates and Least-Square Contrast on One Trial

We first propose a conditional intensity candidate. So for any f∈H with

$$\begin{array}{rcl} H & = & {\left(R\times L_{2}{\left([0,A]\right)}^{M}\right)}^{M}\\ & = & \left\{f=\left({\left(\mu^{\left(m\right)},{\left(g_{\ell }^{\left(m\right)}\right)}_{\ell =1,\ldots ,M}\right)}_{m=1,\ldots ,M}\right):g_{\ell }^{\left(m\right)}\text{ with support in }(0,A\right]\\ & & \text{ and }{∥f∥}^{2}=\sum_{m}{\left(\mu^{\left(m\right)}\right)}^{2}+\sum_{m}\sum_{\ell }\int_{0}^{A}g_{\ell }^{\left(m\right)}{\left(t\right)}^{2}dt<\infty \}, \end{array}$$

we consider the predictable transformation $\psi (f)=(\psi^{\left(1\right)}(f),\ldots ,\psi^{\left(M\right)}(f))$ such that

(18) $$\forall t>0,\;\psi_{t}^{\left(m\right)}(f)=\mu^{\left(m\right)}+\sum_{\ell =1}^{M}\int_{-\infty }^{t-}g_{\ell }^{\left(m\right)}(t-u)dN_{\ell }(u).$$

Note that $\lambda^{\left(m\right)}={\left[\psi^{\left(m\right)}(f^{*})\right]}_{+}$. Therefore, for each *m*, $\psi^{\left(m\right)}(f)$ can be considered as a good intensity candidate as long as it is close enough to the conditional intensity $\lambda^{\left(m\right)}$ (even if $\psi^{\left(m\right)}(f)$ takes negative values). We measure the distance between ψ(f) and *λ* by using the classical $L_{2}$-norm ∥⋅∥:

(19) $$\begin{array}{rcl} {∥\psi (f)-\lambda ∥}^{2} & = & \sum_{m=1}^{M}\int_{T_{1}}^{T_{2}}{\left[\psi_{t}^{\left(m\right)}(f)-\lambda^{\left(m\right)}(t)\right]}^{2}dt\\ & = & \sum_{m=1}^{M}\int_{T_{1}}^{T_{2}}{\left[\psi_{t}^{\left(m\right)}(f)-{\left[\psi_{t}^{\left(m\right)}\left(f^{*}\right)\right]}_{+}\right]}^{2}dt. \end{array}$$

Depending on $f^{*}$, the right-hand side is not observable. But minimizing the last expression with respect to *f* is equivalent to minimizing $f\mapsto \tilde{\gamma }(f)$ with

$$\tilde{\gamma }(f)=-2\sum_{m=1}^{M}\int_{T_{1}}^{T_{2}}\psi_{t}^{\left(m\right)}(f)\lambda^{\left(m\right)}(t)dt+\sum_{m=1}^{M}\int_{T_{1}}^{T_{2}}{\left[\psi_{t}^{\left(m\right)}(f)\right]}^{2}dt.$$

But by definition of the conditional intensity, $\tilde{\gamma }(f)$ is close to γ(f) with

(20) $$\gamma (f)=-2\sum_{m=1}^{M}\int_{T_{1}}^{T_{2}}\psi_{t}^{\left(m\right)}(f)dN^{\left(m\right)}(t)+\sum_{m=1}^{M}\int_{T_{1}}^{T_{2}}{\left[\psi_{t}^{\left(m\right)}(f)\right]}^{2}dt,$$

which is called the least-square contrast. This expression is observable and can be minimized if *f* is parameterized by a fixed number of parameters.

One particular parameterization, that is used in practice, is obtained when each function $g_{\ell }^{\left(m\right)}$ is a piecewise constant function written as

(21) $$g_{\ell }^{\left(m\right)}=\sum_{k=1}^{K}a_{m,\ell ,k}\delta^{-1/2}1_{\left((k-1)\delta ,k\delta \right]},$$

where δ>0 is the size of the bin and *K* the number of bins. So we have Kδ=A. The $a_{m,\ell ,k}$’s are the renormalized coefficients of $g_{\ell }^{\left(m\right)}$ on the regular partition of size *K*. Since $f\to \psi^{\left(m\right)}(f)$ is linear, one obtains

$$\forall t>0,\;\psi_{t}^{\left(m\right)}(f)=\mu^{\left(m\right)}+\sum_{\ell =1}^{M}\sum_{k=1}^{K}a_{m,\ell ,k}\delta^{-1/2}N^{\left(\ell \right)}\left([t-k\delta ,t-(k-1)\delta \right)),$$

still for $f=({\left(\mu^{\left(m\right)},{\left(g_{\ell }^{\left(m\right)}\right)}_{\ell =1,\ldots ,M}\right)}_{m=1,\ldots ,M})$. Let us denote by $a^{\left(m\right)}$ the column vector such that

(22) $${\left(a^{\left(m\right)}\right)}^{\prime }=\left(\mu^{\left(m\right)},a_{m,1,1},\ldots ,a_{m,1,K},a_{m,2,1},\ldots ,a_{m,M,K}\right),$$

where ′ denotes the transpose. Then one can write

(23) $$\forall t>0,\;\psi_{t}^{\left(m\right)}(f)={\left({\mathrm{Rc}}_{t}\right)}^{\prime }a^{\left(m\right)},$$

with ${\mathrm{Rc}}_{t}$ being the renormalized instantaneous counts given by

$${\left({\mathrm{Rc}}_{t}\right)}^{\prime }=\left(1,\delta^{-1/2}{\left(c_{t}^{\left(1\right)}\right)}^{\prime },\ldots ,\delta^{-1/2}{\left(c_{t}^{\left(M\right)}\right)}^{\prime }\right),$$

and with $c_{t}^{\left(\ell \right)}$ being the vector of instantaneous counts with delay of $N^{\left(\ell \right)}$, i.e.,

$${\left(c_{t}^{\left(\ell \right)}\right)}^{\prime }=\left(N^{\left(\ell \right)}\left([t-\delta ,t)\right),\ldots ,N^{\left(\ell \right)}\left([t-K\delta ,t-(K-1)\delta \right)\right)).$$

Hence, by (23), proposing $\psi_{t}^{\left(m\right)}(f)$ as a candidate for the intensity $\lambda^{\left(m\right)}$ of $N^{\left(m\right)}$ amounts to proposing a linear combination of instantaneous counts with delay to model the probability of the next occurrence of a point in $N^{\left(m\right)}$.

Now, minimizing γ(f) over such piecewise constant functions is equivalent, by linearity, to minimizing

$$\gamma (f)=\sum_{m=1}^{M}\left(-2{\left(a^{\left(m\right)}\right)}^{\prime }b^{\left(m\right)}+{\left(a^{\left(m\right)}\right)}^{\prime }Ga^{\left(m\right)}\right)$$

with respect to the vectors $a^{\left(m\right)}$. The vector $b^{\left(m\right)}$ is observable and is given by

$$\begin{array}{rcl} {\left(b^{\left(m\right)}\right)}^{\prime } & = & {\left(\int_{T_{1}}^{T_{2}}{\mathrm{Rc}}_{t}dN^{\left(m\right)}(t)\right)}^{\prime }\\ & = & \left(N^{\left(m\right)}\left([T_{1},T_{2}]\right),\delta^{-1/2}n_{m,1}^{\prime },\ldots ,\delta^{-1/2}n_{m,M}^{\prime }\right), \end{array}$$

where

$$n_{m,\ell }={\left(\int_{T_{1}}^{T_{2}}N^{\left(\ell \right)}\left([t-k\delta ,t-(k-1)\delta \right))dN^{\left(m\right)}(t)\right)}_{k=1,\ldots ,K}$$

and

$$G=\int_{T_{1}}^{T_{2}}{\mathrm{Rc}}_{t}{\left({\mathrm{Rc}}_{t}\right)}^{\prime }dt.$$

Note that the *k*th component of $n_{m,\ell }$ is the number of couples (x,y) with $x\in N^{\left(m\right)}\cap [T_{1},T_{2}]$, $y\in N^{\left(\ell \right)}$ and (y−x)∈((k−1)δ,kδ] and **G** is a symmetric matrix of size 1+MK whose components are the integrated covariations of the renormalized instantaneous counts. The solution of this minimization problem is easily available: If **G** is invertible,

(24) $$\forall m=1,\ldots ,M,\;{\hat{a}}^{\left(m\right)}=G^{-1}b^{\left(m\right)}.$$

Heuristic arguments show that (24) is a natural expression. We can indeed informally write for any *m* that

$$dN^{\left(m\right)}(t)≃\lambda^{\left(m\right)}(t)dt+\text{noise}≃\psi_{t}^{\left(m\right)}\left(f^{*}\right)dt+\text{noise},$$

assuming that at time *t*, the intensity is strictly positive. By linearity of $\psi^{\left(m\right)}$, one can also write that

$$dN^{\left(m\right)}(t)≃{\left({\mathrm{Rc}}_{t}\right)}^{\prime }a_{*}^{\left(m\right)}+\text{noise},$$

where $a_{*}^{\left(m\right)}$ are the coefficients corresponding to $f^{*}$, assuming that $f^{*}$ can be coded in this way. Finally, we obtain

$$\int_{T_{1}}^{T_{2}}{\mathrm{Rc}}_{t}dN^{\left(m\right)}(t)=b^{\left(m\right)}≃\int_{T_{1}}^{T_{2}}{\mathrm{Rc}}_{t}{\left({\mathrm{Rc}}_{t}\right)}^{\prime }a_{*}^{\left(m\right)}dt+\text{noise}≃Ga_{*}^{\left(m\right)}+\text{noise},$$

showing that the estimate given in (24) should be a convenient preliminary estimate.

#### 3.3.2 Least-Square Estimates on Several Trials and Connections with JPSTH and Cross-Correlograms

We observe now *n* trials and, therefore, we have access to ${\left(N_{i}^{\left(1\right)},\ldots ,N_{i}^{\left(M\right)}\right)}_{i=1,\ldots ,n}$ an i.i.d. sample of a multivariate point process on $\left[T_{1},T_{2}\right]$. Each trial has its own history. So to each trial *i*, we can associate as in the previous subsection the matrix **G**, the vectors $b^{\left(m\right)}$ and so on. Depending on the trial *i*, we denote them $G^{\left(i\right)}$, $b^{\left(m,i\right)}$ and so on. The least-square contrast for these n×M spike trains can then be written as

(25) $$\gamma_{n}(f)=\sum_{m=1}^{M}\left(-2{\left(a^{\left(m\right)}\right)}^{\prime }\left(\sum_{i=1}^{n}b^{\left(m,i\right)}\right)+{\left(a^{\left(m\right)}\right)}^{\prime }\left(\sum_{i=1}^{n}G^{\left(i\right)}\right)a^{\left(m\right)}\right)$$

whose solution is given by

(26) $$\forall m=1,\ldots ,M,\;{\hat{a}}^{\left(m\right)}={\left(\sum_{i=1}^{n}G^{\left(i\right)}\right)}^{-1}\left(\sum_{i=1}^{n}b^{\left(m,i\right)}\right).$$

**Fig. 3:**
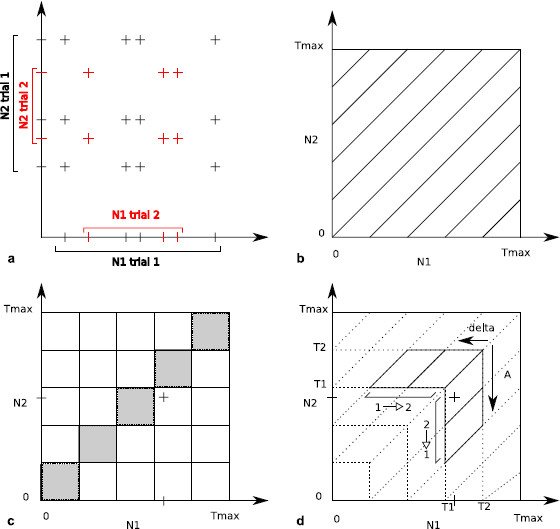
Scatter diagrams and histograms. In panel **a**, we recall how a scatter diagram is constructed for each trial (here *black crosses* for trial 1 and *red crosses* for trial 2) and then superposed. Various histograms can be built with those points, using different partitions. Panel **b** gives the partition corresponding to the classical cross-correlogram. Panel **c** gives the partition of the JPSTH. *The diagonal squares are filled in gray*: if a point of the scatter diagram lies in one of those square, its coordinates correspond to spikes that are very close to each other. Two very close spikes (one on N1, the other one on N2) and their corresponding point in the scatter diagram are added, showing that the reverse is wrong. Therefore, there are some couples that are close to each other and not counted by the JPSTH as such. Panel **d** gives the partition used for the computation of the vectors ${\bar{n}}_{m,\ell }$ of dimension *K* (K=2 here). More precisely, ${\bar{n}}_{1,2}$ corresponds to the vertical part 2→1, whereas ${\bar{n}}_{2,1}$ corresponds to the horizontal part 1→2. The same spikes are added, and they are now counted as close to each other

The quantity $\left(\sum_{i=1}^{n}b^{\left(m,i\right)}\right)$ can be reinterpreted in terms of cross-correlograms and joint-PSTH, following [59]. Indeed we can write

$${\left(\sum_{i=1}^{n}b^{\left(m,i\right)}\right)}^{\prime }=\left({\left[N^{\left(m\right)}\right]}^{a,n}\left([T_{1},T_{2}]\right),\delta^{-1/2}{\bar{n}}_{m,1}^{\prime },\ldots ,\delta^{-1/2}{\bar{n}}_{m,M}^{\prime }\right),$$

where for any *ℓ*,

$${\bar{n}}_{m,\ell }=\sum_{i=1}^{n}{\left(\int_{T_{1}}^{T_{2}}N_{i}^{\left(\ell \right)}\left(\left[t-k\delta ,t-(k-1)\delta \right]\right)dN_{i}^{\left(m\right)}(t)\right)}_{k=1,\ldots ,K},$$

and ${\left[N^{\left(m\right)}\right]}^{a,n}$ is the aggregated process over all the *n* trials for the *m*th neuron. The quantity ${\bar{n}}_{m,\ell }$ can be reinterpreted as a particular histogram based on the joint peristimulus time scatter diagram as the JPSTH or the cross-correlogram (see Fig. 1 of [59] and Fig. 3 of the present article). More precisely as detailed in Fig. 3, the counts ${\bar{n}}_{m,\ell }$ are close to a cross correlogram except that representations are not based on squares but on herringbones. Local features are then preserved, as for the JPSTH. Furthermore, the elements of the partition have the same area and can therefore be compared more easily. Besides, for small disjoint intervals $\left[T_{1},T_{2}\right]$ with an increasing parameter *A* (corresponding to the maximal size of the support of the interaction functions), and for *δ* close to 0, we obtain representations close to the JPSTH, except that the limits are not parallel to the axis, but parallel to the diagonal. This change of orientation smooths the binning effects. Indeed the quantity that is binned for the quantities ${\bar{n}}_{m,\ell }$ is the delay itself between two points, whereas for the JPSTH, each position of the points is first binned. Therefore, two points whose distance is less than *δ* are always counted as such in one of the diagonal parallelograms for ${\bar{n}}_{m,\ell }$, whereas they may eventually not be counted in a diagonal square of the JPSTH, because one point appears in one bin and the other one in another bin (see Figs. 3c and 3d). This problem of information loss when binning is involved has already been discussed for the coincidence counts [15]. 

JPSTH and cross correlograms have been used for a long time in neuroscience, without links with any model. The formula (26), for the least-square estimate, shows the link between those descriptive statistics (more precisely the ${\bar{n}}_{m,\ell }$’s) and the parameters of the Hawkes model. To recover the parameters, we need, in particular, to inverse the matrix $\left(\sum_{i=1}^{n}G^{\left(i\right)}\right)$. This matrix quantifies for instance the following situation. Assume that M=3 and that the interaction functions $h_{2}^{\left(1\right)}$ and $h_{3}^{\left(2\right)}$ are large on [0,δ] and null elsewhere. We also assume that all the other interaction functions are null. In this situation, ${\bar{n}}_{1,3}$ (or at least its first coordinate) will be large even if there is no direct interaction from $N_{3}$ on $N_{1}$. The matrix $\left(\sum_{i=1}^{n}G^{\left(i\right)}\right)$ cumulates all these features (and also the fixed effect due to the spontaneous parameter, which needs to be subtracted) and inverting it enables us to find an estimate of the true interactions. See also [58] for a more visual transcription. 

Note, however, that even if many coefficients are null as in the above described situation, due to the random noise, the estimates ${\hat{a}}^{\left(m\right)}$ have non-zero coordinates almost surely. Therefore, it is difficult to interpret the resulting estimate in terms of functional connectivity graph [58]. Moreover, if we wish to capture all the features, it is preferable to take *A* large and *δ* small. Therefore, the number of parameters of the model, depending on $K=A\delta^{-1}$, increases. With a small number of trials *n* and a small interval $\left[T_{1},T_{2}\right]$, the least-square estimate is doomed to be quite poor as the MLE [19]. 

To remedy these problems, we now consider $\ell_{1}$ penalization to find a nonparametric estimate with adaptive properties and prescribed convergence rate.

#### 3.3.3 Lasso Estimate

The Lasso method as developed by [57], is based on the following penalized least-square criterion, reformulated here in the context of *n* i.i.d. trials: for any m=1,…,M,

(27) $$\begin{array}{rcl} {\tilde{a}}^{\left(m\right)} & \in & \arg\min_{a^{\left(m\right)}}(-2{\left(a^{\left(m\right)}\right)}^{\prime }\left(\sum_{i=1}^{n}b^{\left(m,i\right)}\right)\\ & & +{\left(a^{\left(m\right)}\right)}^{\prime }\left(\sum_{i=1}^{n}G^{\left(i\right)}\right)a^{\left(m\right)}+2{\left(d^{\left(m\right)}\right)}^{\prime }\left|a^{\left(m\right)}\right|), \end{array}$$

where $\left|a^{\left(m\right)}\right|$ denotes the vector whose coefficients are the absolute values of the coefficients of $a^{\left(m\right)}$ and where

$${\left(d^{\left(m\right)}\right)}^{\prime }=(d_{m,0},d_{m,1,1},\ldots ,d_{m,1,K},d_{m,2,1},\ldots ,d_{m,M,K})$$

is a vector of positive observable weights given by

(28) $$d_{m,\ell ,k}=\sqrt{2\gamma \ln\left(n(T_{2}-T_{1})\right){\hat{V}}_{m,\ell ,k}}+\frac{\gamma \ln(n(T_{2}-T_{1}))}{3}{\hat{B}}_{\ell ,k},$$

where

$$\begin{array}{rcl} {\hat{V}}_{m,\ell ,k} & = & \sum_{i=1}^{n}\int_{T_{1}}^{T_{2}}\delta^{-1}{\left[N_{i}^{\left(\ell \right)}\left([t-k\delta ,t-(k-1)\delta \right))\right]}^{2}dN_{i}^{\left(m\right)}(t),\\ {\hat{B}}_{\ell ,k} & = & \delta^{-1/2}\underset{i,t\in [T_{1},T_{2}]}{\sup}N_{i}^{\left(\ell \right)}\left([t-k\delta ,t-(k-1)\delta \right)), \end{array}$$

and with

$$d_{m,0}=\sqrt{2\gamma \ln\left(n(T_{2}-T_{1})\right){\left[N^{\left(m\right)}\right]}^{a,n}\left([T_{2},T_{1}]\right)}+\frac{\gamma \ln(n(T_{2}-T_{1}))}{3}.$$

Since the criterion (27) is convex, the minimization problem can be performed quite easily. The function f∈H associated with $\tilde{a}$ is denoted ${\hat{f}}^{B}$, in reference to the Bernstein inequality that governs the shape of the weights (see [57]). 

Because the penalty term added to the least-square criterion is a weighted $\ell_{1}$-norm, the resulting estimate is sparse and many coordinates in ${\tilde{a}}^{\left(m\right)}$ will be null (see [60] for the seminal paper on Lasso methods). This estimate and much more general forms have been studied quite intensively in [57]. In Additional File 3, we prove an oracle inequality for a slight modification of the present estimate, whose exact form can also be found in [58]. 

Let us just present the result informally to highlight the main properties (the complete version can be found in Additional File 3). An oracle inequality, in the same spirit as Theorem 2, is proved. The main difference is that it holds on a event with large probability and not in expectation. We have an upper bound of

(29) $$\sum_{i}\sum_{m}\int_{T_{1}}^{T_{2}}{\left(\psi^{\left(m\right)}{\left({\hat{f}}^{B}\right)}_{i}(t)-\lambda_{i}(t)\right)}^{2}dt,$$

that constitutes a compromise, as usual, between a bias term and a variance term. Minimizing the bias gives the best linear approximation of *λ* of the form ψ(f) and this even if *λ* is not of the form ψ(f). In this sense, it applies in particular to Hawkes processes with self-inhibition (i.e., negative $h_{m}^{\left(m\right)}$’s), which models refractory periods [22] and for which $f\to \lambda ={\left(\psi (f)\right)}_{+}$ is not linear anymore. Finally, (29) leads to a control of the left-hand side of (8) adapted to the context of this section. Under further technical assumptions, we can then prove that Test 4 can be applied. We refer the reader to [57] for more details that are omitted here to avoid too tedious technical aspects. 

The last point already developed in [57] is that Lasso estimates are most of the time biased in practice. To overcome this problem, a two step procedure is proposed. It consists in finding the non zero coefficients of ${\hat{f}}^{B}$ and performing a classical least-square estimate on this support. We denote this two-step estimate ${\hat{f}}^{\mathrm{BO}}$.

## 4 Practical Performance

### 4.1 Description of the Data

#### 4.1.1 Real Data

The data used here are a small subset of already partially published data in previous experimental studies [15,22,61,62]. These data were collected on a 5-year-old male rhesus monkey who was trained to perform a delayed multidirectional pointing task. The animal sat in a primate chair in front of a vertical panel on which seven touch-sensitive light-emitting diodes were mounted, one in the center and six placed equidistantly (60 degrees apart) on a circle around it. The monkey had to initiate a trial by touching and then holding with the left hand the central target. After a delay of 500 ms, the preparatory signal (PS) was presented by illuminating one of the six peripheral targets in green. After a delay of either 600 or 1200 ms, selected at random with various probability, it turned red, serving as the response signal and pointing target. During the first part of the delay, the probability for the response signal to occur at 500+600ms=1.1s was 0.3. Once this moment passed without signal occurrence, the conditional probability for the signal to occur at 500+600+600ms=1.7s changed to 1. The monkey was rewarded by a drop of juice after each correct trial, i.e., a trial for which the monkey touches the correct target at the correct moment.

Signals recorded from up to seven independently moving microelectrodes (quartz insulated platinum–tungsten electrodes, impedance: 2–5 MO at 1000 Hz) were amplified and band-pass filtered from 300 Hz to 10 kHz. Single unit activity was obtained by performing an online discrimination of spikes on each electrode. Spikes were firstly selected by taking into account their amplitude using an online window discriminator with high-pass and low-pass filters. In cases where spikes were not discriminable due to their amplitude only, the electrode was moved until the signals were sufficiently distinct to be discriminable on this basis. Although off-line spike sorting was available, it was not used in this study. Indeed, beyond the reservations that one may have concerning the variable quality of the output of such software, the use of clean original electrophysiological signals makes safer the more specific study of precise neuronal synchronization. Neuronal data along with behavioral events (occurrences of signals and performance of the animal) were stored on a PC for off-line analysis with a time resolution of 1 kHz.

**Fig. 4:**
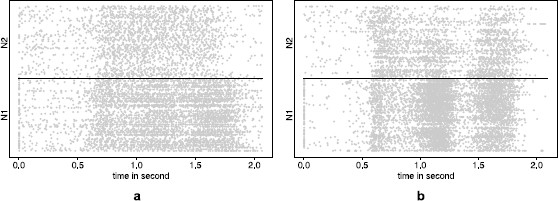
Raster plots of the data sets. In panel **a** the rasters associated to Data Set **A** i.e. (N1A, N2A). In panel **b**, the ones associated to Data Set **B**, i.e. (N1B, N2B). Each *line* corresponds to a trial, each *dot* to a spike

Two sets of data are here considered. They both correspond to a probability of 0.3 that the response signal occur at 1.1 s for the monkey, but only correct trials where the response signal occurs at 1.7 s are considered. On both data sets, two neurons have been recorded simultaneously over $\left[0,T_{\max}\right]$ where $T_{\max}$ is approximately two seconds. In the sequel, Data Set **A** (respectively **B**) corresponds to the pair of neurons (N1A, N2A) (respectively to the pair of neurons (N1B, N2B)). In Data Set **A** (respectively **B**), 177 trials (respectively 141 trials) are considered. Figure 4 plots the rasters associated with both data sets. However, because 6 different directions of movement were proposed to the monkey, we can also consider in both data sets, 6 subsets of trials, each subset corresponding to a prescribed direction of movement (see Table 1 for a repartition of the number of trials per direction). 

**Table 1:** Repartition of number of trials on the real data sets

	Direction						Total
	1	2	3	4	5	6	
Data Set **A**	28	31	30	35	28	25	177
Data Set **B**	23	24	26	18	30	20	141

Therefore, *n*, the total number of trials will be close to 200 if one aggregates over all the directions or will belong to the interval [20,35] if one considers the trials according to the directions. Those trials are assumed to be i.i.d. This assumption is more reasonable if one considers trials for a fixed given direction.

#### 4.1.2 Simulated Data

To assess the performance of our procedure, simulated data for which the underlying model is known have also been simulated. Three different data sets have been simulated, with the thinning method [63]: 

• (S-HomPoi) Spikes are distributed according to an homogeneous Poisson processes of intensity 20 Hz on [0,2] s.

• (S-InPoi) Spikes are distributed according to an inhomogeneous Poisson processes with piecewise continuous intensity on [0,2] s given by

$$t\to \lambda (t)=\sum_{i=1}^{3}\left[g_{i}+h_{i}e^{-4*{\left(t-c_{i}\right)}^{2}/(r_{i}^{2}-{\left(t-c_{i}\right)}^{2})}\right]1_{t\in [c_{i}-r_{i};c_{i}+r_{i})},$$

with g=[5,30,0], h=[12.5,15,12.5], c=[0.375,1.25,1.825], and r=[0.375,0.5,0.125].

• (S-Haw) Two spike trains are simulated according to a bivariate Hawkes process observed on [0,2]. Each process is respectively denoted $N^{\left(1\right)}$ and $N^{\left(2\right)}$. Their intensities are given by (17) with spontaneous parameters $\nu^{\left(1\right)}=\nu^{\left(2\right)}=20$ Hz and interaction functions $h_{1}^{\left(1\right)}=h_{2}^{\left(2\right)}=-20\times 1_{\left[0,0.005\right]}$, $h_{2}^{\left(1\right)}=60\times 1_{\left[0,0.01\right]}$ and $h_{1}^{\left(2\right)}=0$.

 Each time a *n* i.i.d. sample is drawn.

The several treatments have been done in R except Willett and Nowak’s estimate (WN) for which Matlab has been used.

### 4.2 Results

#### 4.2.1 Checking the Homogeneous Poisson Assumption

One of the simplest tests is to check whether the number of spikes per trial obeys a Poisson distribution with unknown parameter. Since the Poisson distribution is discrete, one can use a chi-square test with one estimated parameter, whose results are summarized in Table 2. Since the number of spikes per trial is also a Poisson variable for inhomogeneous Poisson processes, it is reasonable to have large *p*-values for (S-HomPoi) and (S-InPoi). Relatively smaller *p*-values appear for (S-Haw), but they are not small enough for a clear rejection: This test seems therefore not very powerful. Note that a very close test has been used in [8] on disjoint intervals of observations. The estimate of the parameters were computed via a prescribed parametric model and, therefore, the procedure was testing both the Poisson assumption and the parametric assumption. To our knowledge, chi-square tests cannot be adapted to a nonparametric plug-in. On Data Sets **A** and **B**, the most undoubtedly rejection appears for the pooled data, which can be explained by the fact that those data are not i.i.d. 

**Table 2:** *p*-values of the chi-square test of the Poissonian distribution for the number of spikes per trial. The following code is used: ∘ corresponds to a *p*-value of the test by upper values in $\left[10^{-2},10^{-1}\right)$, ▵ to a *p*-value of the test by upper values in $\left[10^{-3},10^{-2}\right)$, ▵▵ to a *p*-value of the test by upper value in $\left[10^{-4},10^{-3}\right)$, ▵▵▵ to a *p*-value of the test by upper value in $\left(-\infty ,10^{-4}\right)$. The signs are filled in black if the *p*-values correspond to rejection of a Benjamini and Hochberg (BH) multiple test method [64] either on the simulated data (left part of the table) or on Data Sets **A** and **B** (right part of the table)

	*n*			Directions						Pooled
	40	200		1	2	3	4	5	6	
S-HomPoi			N1A	∘		∘	•	▲		▲▲▲
S-InPoi			N2A				▲▲	∘	∘	▲▲▲
S-Haw ($N^{\left(1\right)}$)			N1B	∘	∘		∘			▲▲▲
S-Haw ($N^{\left(2\right)}$)	∘		N2B	∘	•					▲▲▲

The second simplest test (as prescribed by Yana et al. [1] for instance) is the classical Kolmogorov–Smirnov test of uniformity performed on all the spikes, once all the trials have been aggregated, which relies on the fact that conditionally to the total number of observed spikes the points of a homogeneous Poisson process should obey a uniform distribution. Table 3 shows the corresponding *p*-values. It is quite coherent to have large *p*-values for (S-HomPoi) and very small *p*-values for (S-InPoi) because of the lack of stationarity of the later. Since (S-Haw) is stationary, aggregation dilutes the dependence and it explains that this test is not powerful and that the *p*-values are large in this case. This test clearly rejects for Data Sets **A** and **B** the homogeneous Poisson hypothesis. 

**Table 3:** *p*-values of the classical K.S. test of uniformity on the spikes, all trials being aggregated. Same codes as in Table 2. Note that none of the *p*-values were close enough to 1 to force a rejection by the test by lower values (i.e., rejection when the test statistic is smaller than $k_{n,\alpha }$, which corresponds to *p*-values of the test by upper values larger than 1−α)

	*n*			Directions						Pooled
	40	200		1	2	3	4	5	6	
S-HomPoi			N1A	▲▲▲	▲▲▲	▲▲▲	•	▲▲▲	▲▲	▲▲▲
S-InPoi	▲▲▲	▲▲▲	N2A	▲▲▲	▲▲▲	▲▲▲	▲▲▲	▲▲▲	▲▲▲	▲▲▲
S-Haw ($N^{\left(1\right)}$)			N1B	▲▲▲	▲▲▲	▲▲▲	▲▲▲	▲▲▲	▲▲▲	▲▲▲
S-Haw ($N^{\left(2\right)}$)			N2B	▲▲▲	▲▲▲	▲▲▲	▲▲▲	▲▲▲	▲	▲▲▲

Another test of first-order statistics as explained in [1] is the exponential test on the ISI (see Table 4). Here, we apply our version of the exponential test, i.e., Test 1, using the subsampling scheme. As soon as there is enough trials, this test is powerful enough to detect the nonstationarity (S-InPoi), but also the dependence (S-Haw), since there is no aggregation to dilute the dependence between consecutive points in one trial with respect to the previous test. On Data Sets **A** and **B**, the result is almost the same as the previous test. 

**Table 4:** *p*-values of Test 1 on the ISI, with a subsample size $\left\lfloor {\left[n_{\mathrm{tot}}^{\mathrm{ISI}}\right]}^{2/3}\right\rfloor$, where $n_{\mathrm{tot}}^{\mathrm{ISI}}$ is the total number of ISI that have been observed in all the trials. Same codes as in Table 2 for the *p*-values. Note that none of the *p*-values were close enough to 1 to force a rejection by the test by lower values (i.e., rejection when the test statistic is smaller than $k_{n,\alpha }$, which corresponds to *p*-values of the test by upper values larger than 1−α)

	*n*			Directions						Pooled
	40	200		1	2	3	4	5	6	
S-HomPoi			N1A	▲▲▲	▲▲▲	▲	•		∘	▲▲▲
S-InPoi	▲▲	▲▲▲	N2A	▲▲▲	▲▲▲	▲▲▲	▲	▲▲▲	▲▲▲	▲▲▲
S-Haw ($N^{\left(1\right)}$)	∘	▲	N1B	▲▲▲	▲▲▲	▲▲▲	•	▲▲▲	▲▲▲	▲▲▲
S-Haw ($N^{\left(2\right)}$)	▲	▲▲▲	N2B	•	▲▲▲	▲	∘	▲▲	▲	▲▲▲

#### 4.2.2 Checking the Inhomogeneous Poisson Assumption

**Fig. 5:**
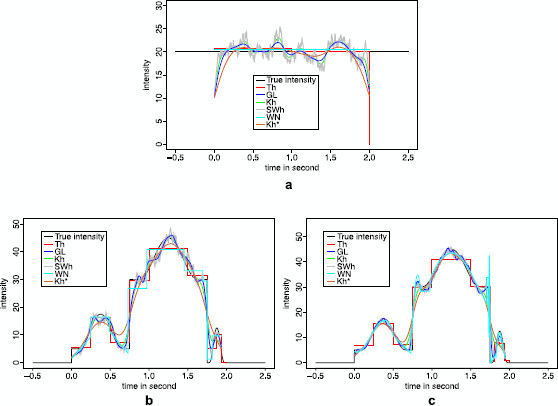
Reconstructions of the intensity *λ* on simulated Poisson data. Reconstructions for (S-HomPoi) and (S-InPoi) over *n* trials, observed on [0,2]. <monospace>Th</monospace> corresponds to the adaptive histogram ${\hat{\lambda }}_{n}^{\mathrm{Th}}$, <monospace>GL</monospace> to the Goldenshluger and Lepski’s method ${\hat{\lambda }}_{n}^{\mathrm{GL}}$ with the Gaussian kernel, <monospace>Kh</monospace> to a fixed bandwidth h=0.05 for the Gaussian kernel, <monospace>SWh</monospace> to the sliding window with h=0.05, <monospace>WN</monospace> to the Willett and Nowak’s method and <monospace>Kh</monospace>* to the rule of the thumb. Panel **a** corresponds to (S-HomPoi) with n=40, panel **b** to (S-InPoi) with n=40 and panel **c** to (S-InPoi) with n=200

Let us first look at the reconstructions of the underlying intensities. We first program two very basic kernel estimates (10): the sliding window (i.e., $K=(1/2)1_{\left[-1,1\right]}$) with length 0.1 s (i.e., bandwidth 0.05), denoted ${\hat{\lambda }}_{n}^{{\mathrm{SW}}_{h}}$, and the Gaussian kernel (used for instance in [8]) with the same bandwidth and denoted ${\hat{\lambda }}_{n}^{K_{h}}$. As [8], we also programmed a data-driven choice of bandwidth, called the thumb rule (here we followed [39] to construct it): it is denoted ${\hat{\lambda }}_{n}^{K_{h^{*}}}$. Finally, we programmed the three presented adaptive method. The GL estimate, ${\hat{\lambda }}_{n}^{\mathrm{GL}}$ is programmed with the Gaussian kernel, with the choice η=0.5 and with the bandwidths family

$$H=\left\{D^{-1}:D=4,5,6,7,8,9,10,11,12,14,16,18,20,22,25,30,35,40,45,50\right\},$$

which has shown a robust behavior on various simulations, for the present considered size of *n* (n≈40). The thresholding estimate on the Haar basis, ${\hat{\lambda }}_{n}^{\mathrm{Th}}$, has been performed with $j_{0}=15$ and γ=1. We use the Matlab package FreeDegree for the Willett and Nowak estimate, ${\hat{\lambda }}_{n}^{\mathrm{WN}}$. Reconstructions for (S-HomPoi) and (S-InPoi) are given in Fig. 5. 

First of all, ${\hat{\lambda }}_{n}^{{\mathrm{SW}}_{h}}$ is clearly the worst choice, as expected for such a rough kernel. Figure 5a shows the reconstruction of a constant intensity. Kernel estimates with Gaussian kernels are oscillating; the thumb rule bandwidth $h^{*}$ is larger than $\hat{h}$, the GL bandwidth. The fixed bandwidth h=0.05 is the smallest one and is quite inadequate in this setting. The adaptive Haar thresholding rule ${\hat{\lambda }}_{n}^{\mathrm{Th}}$ is much better in this case. The WN method is able to reconstruct perfectly the flat line. For (S-InPoi), the intensity has large jumps and smooth bumps (Figs. 5b and 5c). For such an irregular intensity and for a small number of trials (Fig. 5b), the thresholding estimate and the WN method are both able to recover the jumps perfectly but the smooth bumps are estimated by a piecewise constant function. The Gaussian kernels are better for the estimation of the bumps, but of course, they cannot detect the jumps. In this respect, the GL bandwidth is the best, whereas ${\hat{\lambda }}_{n}^{K_{h^{*}}}$ and ${\hat{\lambda }}_{n}^{K_{h}}$ are too smooth. For a large number of trials (Fig. 5c), the thresholding estimate is a bit refined but clearly suffers from a lack of smoothness. Unlike ${\hat{\lambda }}_{n}^{K_{h^{*}}}$ and ${\hat{\lambda }}_{n}^{K_{h}}$, ${\hat{\lambda }}_{n}^{\mathrm{GL}}$ is reconstructing all the three bumps. The WN method is reconstructing more accurately the jumps despite some important boundary artefacts. It also gives smoother reconstructions for the bumps. In conclusion, the GL method clearly gives a bandwidth choice that adapts to high irregularity of the intensity with respect to other choices, whereas the thresholding estimate, which leads to an adaptive histogram, is more spatially adaptive despite its lack of smoothness. Up to boundary effects, the WN methodology seems to be the most accurate, since it adapts to the regularity of the underlying intensity. Note, however, that on an interval with a few number of points, this method provides a piecewise constant reconstruction, even if the underlying intensity is smooth, because this choice is more robust. This conclusion is also coherent with two previous and more extensive studies (see [43,44]). 

**Fig. 6:**
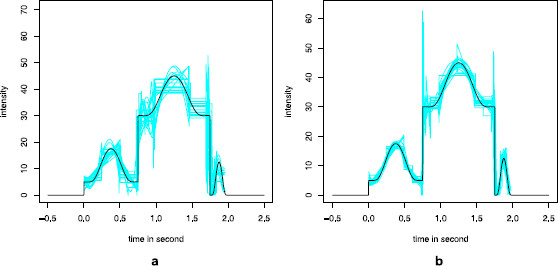
Variability of the reconstructions of the intensity *λ* on simulated Poisson data. Superposition of 20 reconstructions by Willett and Nowak’s method for (S-InPoi) over *n* trials, observed on [0,2]. In panel **a**, n=40. In panel **b**, n=200

To understand more clearly the variability of WN estimates, we have drawn 20 reconstructions in Fig. 6, which confirms that the variability is small and diminishes when *n* grows. The estimates are more likely to be piecewise constant for small *n*. For large *n*, there are some edge effects around the jumps, but the amplitude around the bumps is very small thanks to a smooth estimate in those parts of the curve. 

**Fig. 7:**
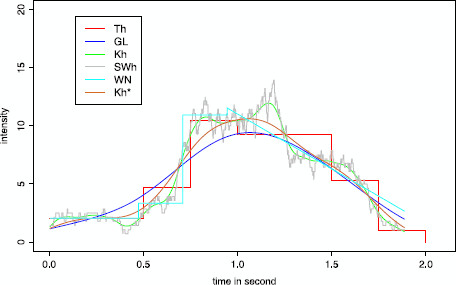
Example of estimation of the intensity *λ* on real data. Estimations for neuron N2A, in direction 1. Same conventions as in Fig. 5

As an example of estimation on real data, Fig. 7 displays an interesting case, namely N2A in direction 1, where one sees clearly that WN estimate is able to find correctly the main jump in the intensity (which is clearly seen in the raster plots of Fig. 4), but also to produce a smoother estimate at some places (here a straight decreasing line on the right) to reproduce the much slower attenuation of the firing rate that can be seen in the raster plots. 

Now let us apply the different proposed tests. First, Test 2 has been applied on the simulated data sets with n=40 and $p=\lfloor n^{2/3}\rfloor$ and nothing was declared significant. However, the *p*-values corresponding to (S-Haw) are abnormally large. To take this into account, we have performed also a variant of Test 2 where one rejects if the same test statistic is now smaller than $k_{n,\alpha }$. This test consists therefore in rejecting the Poisson hypothesis when both estimated distributions are too close. As Test 2, this test is also asymptotically of level *α*, by application of Proposition 2. Actually, all the tests presented here can be said to reject “by upper values” and have therefore a version “by lower values.” On each set (simulated or not) of Test 2 *p*-values (by upper and lower values), one can perform a Benjamini and Hochberg procedure with FDR 5 % [64]. It declares both processes in (S-Haw) as non-Poissonian in the family of simulated data (*p*-values in $\left[10^{-3},10^{-2}\right)$). Due to the high variability of the reconstructions on Data Sets **A** and **B**, which depend on the considered direction, it was not possible to pool the data together and, therefore, the corresponding tests have been performed direction by direction. However, Tests 2 by upper or lower values do not detect anything on Data Sets **A** and **B**.

Tests 3 (by upper or lower values) do not detect anything on the simulated data sets. However, on Data Sets **A** and **B** (see Table 5), Test 3 clearly rejects the Poisson hypothesis for most of the directions of N1B and in this sense, Test 3 is more powerful than Test 2. 

**Table 5:** *p*-values of Test 3 on Data Sets **A** and **B**, with a subsample size $\left\lfloor n^{2/3}\right\rfloor$, where *n* is the number of trials. Same codes as Table 2 for the *p*-values of the test by upper values. For the *p*-values of the test by lower values, same codes except that ∘ becomes □ and ▵ becomes ▽

		Directions					
		1	2	3	4	5	6
N1A	Th		□				
	GL						
	WN						
N2A	Th						
	GL						
	WN						
N1B	Th			▵	∘		
	GL	▲▲	▲▲▲	▲▲▲	▲▲		▲
	WN			▵			
N2B	Th	▵	∘				□
	GL	∘		∘	∘	∘	
	WN	∘					

Test 5 is, as expected, more powerful than Tests 2 and 3 and detects the non-Poissonian structure in (S-Haw) (see Table 6). More importantly, on Data Sets **A** and **B**, all the *p*-values of Test 5 (by upper values) are smaller than 10^−14^ making clear that those data are not inhomogeneous Poisson processes. 

**Table 6:** *p*-values of Test 5 on the simulated data sets, with n=40 and a subsample size $\left\lfloor n^{2/3}\right\rfloor$. Same codes as in Tables 2 and 5 for the *p*-values

	Th	GL	WN
S-HomPoi			
S-InPoi			
S-Haw ($N^{\left(1\right)}$)	▲	▲	▲
S-Haw ($N^{\left(2\right)}$)	∘	∘	∘

#### 4.2.3 Checking the Hawkes Assumption

**Fig. 8:**
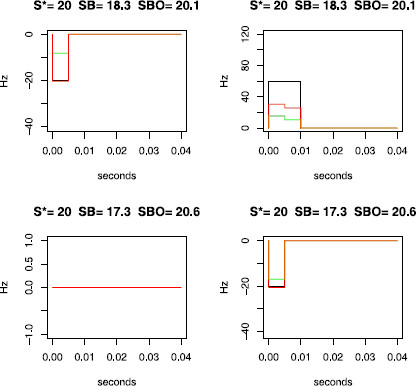
Reconstruction of the interaction functions on simulated data. Reconstructions for (S-Haw) with n=40 trials, with $T_{1}=0.05$ and $T_{2}=2$. *On the upper left*, the interaction function $h_{1}^{\left(1\right)}$, *on the upper right*, $h_{2}^{\left(1\right)}$, *on the bottom left*$h_{1}^{\left(2\right)}$ and *on the bottom right*$h_{2}^{\left(2\right)}$. *In black*, the true interaction functions, *in green*, reconstruction by method ${\hat{f}}^{B}$, *in red*, reconstruction by method ${\hat{f}}^{\mathrm{BO}}$. *On the top of each graphics*, the true spontaneous parameter $\nu^{\left(1\right)}$ on the top and the true parameter $\nu^{\left(2\right)}$ on the bottom are referred by <monospace>S</monospace>*. The estimated spontaneous parameters by method ${\hat{f}}^{B}$ are referred as <monospace>SB</monospace> and the estimated spontaneous parameters by method ${\hat{f}}^{\mathrm{BO}}$ are referred as <monospace>SBO</monospace>. We used K=8, δ=0.005, and γ=1

**Fig. 9:**
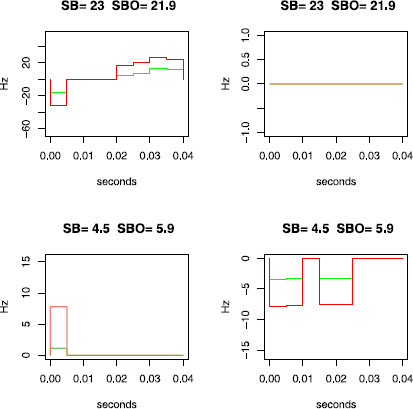
Example of estimation of the interaction functions on real data. Estimations for Data Set **A** in direction 2 with n=40 trials, with $T_{1}=0.05$ and $T_{2}=2$. Same conventions as in Fig. 8

Once again, Data Sets **A** and **B** have to be treated direction by direction because of the high variability of the reconstruction. However, because of the very small number of trials per direction, it was not possible to look at very small subintervals $\left[T_{1},T_{2}\right]$. Therefore, we decided to look at the largest interval that one can take i.e. [Kδ,2]. We choose K=8, δ=0.005 and γ=1. On simulated data (Fig. 8), ${\hat{f}}^{B}$ and ${\hat{f}}^{\mathrm{BO}}$ recover the support of the interaction functions and also find that $h_{1}^{\left(2\right)}=0$, which could not have been possible with a classical least-square estimate (see also more comments on the functional connectivity graph in [58]). Moreover, ${\hat{f}}^{\mathrm{BO}}$ is less biased than ${\hat{f}}^{B}$ as expected. We provide one estimation of the interaction functions for Data Set **A** in direction 2 (Fig. 9), where it clearly appears a one way excitation of N2A on N1A. This is coherent with a previous study on the same data set, which finds this pair of neurons dependent through a complete different method [22]. Note also that at short range the self-interaction functions are negative, fact which is consistent with refractory periods. 

Test 4 is used to check the Hawkes assumption, with $\hat{f}$ given either by ${\hat{f}}^{B}$ or ${\hat{f}}^{\mathrm{BO}}$ (see Table 7). It is coherent to find that, on simulated data, the Hawkes assumption is accepted for (S-Haw), but also for (S-HomPoi), which is a particular case of Hawkes process with null interaction functions. On the contrary, Test 4 detects that (S-InPoi) is not a Hawkes process. On Data Sets **A** and **B**, the Hawkes assumption is sometimes accepted on the whole interval of observations. Let us focus for instance on N1A in direction 2 (see also Fig. 9). This process could not be a Poisson process, since the *p*-value of Test 5 is smaller than 10^−14^. However, the large *p*-value for Test 4 combined with Fig. 9 means that this process can be explained via a pure self-interaction process, which is negative at short range (refractory period) and positive at larger range. Note that 9 processes on the 24 are not explained in this way (in particular N2A in direction 2), and are not Poisson either: Ihis can be due to a too large lack of stationarity, combined with a large dependence between points. 

**Table 7:** *p*-values of Test 4 with a subsample size $\left\lfloor n^{2/3}\right\rfloor$. Same codes as in Tables 2 and 5 for the *p*-values

		*n* = 40			Directions					
					1	2	3	4	5	6
S-HomPoi	B		N1A	B			∘	∘	∘	
	BO	∘		BO					▲▲▲	∘
S-InPoi	B	▲	N2A	B	∘	▲▲		▲		∘
	BO			BO	∘					∘
S-Haw ($N^{\left(1\right)}$)	B		N1B	B		▵	▲		∘	
	BO			BO						
S-Haw ($N^{\left(2\right)}$)	B		N2B	B	▲▲▲	▲▲▲	∘		▲	
	BO	∘		BO		∘	▲	▲		

## 5 Conclusion

When using the time-rescaling theorem to assess whether an observed spike train obeys a certain probabilistic model (e.g., Poisson, Hawkes, etc.), a plug-in step is currently performed [1-3,8,24]. If this plug-in step is done without care the resulting test may be much too conservative leading to poor detections (see Fig. 1). We propose here to use the subsampling as an almost universal solution when dealing with Kolmogorov–Smirnov tests of uniformity, such a universal solution being completely new. The main requirement is to have access to an estimate of the underlying intensity, whose rate of convergence is known (see, for instance, (5)).

In classical previous works such as [8,23], parametric estimates such as MLE over a prescribed parametric model are used as plug-in estimates, which are converging toward the true intensity when the underlying parametric model is true. Therefore, when this parametric plug-in is used inside omnibus tests (and in particular the ones presented here), one has to be careful that we are not only testing the probabilistic assumption, but also the fact that the intensity belongs to the parametric model. To overcome this problem, we advertise for the use of nonparametric estimates and more precisely to adaptive estimates, for which rates of convergence are known under lighter assumptions on the intensity than a prescribed parametric assumption. Moreover, those adaptive methods have very good practical performance making them also very good in practice for estimation. 

On some simulated data and on some real data sets, we have shown that our method performs very well. However, there are still two main directions in which our work need to be pursued in order to provide a more complete answer on real data sets. First of all, the KS test of exponentiality on the ISI can also be performed instead of the KS test of uniformity [1]. If the method with subsampling can be adapted to this case, we have presently no guarantee that this test would have a controlled level. Indeed we have no equivalent of Proposition 2 or Theorem 1 for the ISI repartition. The second main drawback is that we are clearly able to reject (at least on the presented real data sets) both homogeneous and inhomogeneous Poisson assumptions. We are also able to test whether the processes are Hawkes or not, which in particular takes into account refractory periods and dependence between several spike trains. However, the Hawkes model reflects stationary features, and cannot model nonstationary data. Therefore, we would need a more general model, which includes the dependence as in the Hawkes model presented here, but also the non- stationarity as in the inhomogeneous Poisson process. At the present moment, models reflecting both are not compatible with a full agnostic approach where no assumption is made on the underlying functions, the estimation problem being not completely identifiable [16]. A first step will be therefore to provide a trade off between estimation capacity and not too restrictive assumptions on the process itself, with respect to real spike train data. 

## Electronic Supplementary Material

## Competing Interests

The authors declare that they have no competing interests.

## Authors’ Contributions

CTM is the main initiator of the project both from a theoretical and practical point of view and did most of the numerical experiments. PRB participated in both theoretical and practical aspects and did most of the redaction. VR participated in both theoretical aspects and redaction. FG highlighted the main questions arising in neuroscience, thanks to his experience in this field.

## Supplementary Material

Additional file 1 Kolmogorov–Smirnov tests, plug-in and sub-sampling: the proof of the main results in Sect. 2 (PDF 132 KB) Click here for file

Additional file 2 Proof of Theorem 2 (PDF 94.6 KB) Click here for file

Additional file 3 Adaptive properties of the Lasso estimate for Hawkes processes: the oracle inequality satisfied by the Lasso estimate ${\hat{f}}^{B}$ (see Sect. 3.3.3) (PDF 84.5 KB) Click here for file
