# Supplementary material for: Goodness-of-Fit Tests and Nonparametric Adaptive Estimation for Spike Train Analysis
Source: J Math Neurosci. 2014 Apr 17;4:3. doi: 10.1186/2190-8567-4-3 (PMC4021619; doi:10.1186/2190-8567-4-3)
Supplement: Additional file 1 — Kolmogorov–Smirnov tests, plug-in and sub-sampling: the proof of the main results in Sect. 2 (PDF 132 KB) [file 2190-8567-4-3-S1.pdf]

Additional File 1 of "Goodness-of-fit tests and non-parametric adaptive estimation for spike train analysis": Kolmogorov-Smirnov tests, plug-in and sub-sampling

Patricia Reynaud-Bouret*[1] and Vincent Rivoirard[2] and Franck Grammont[1] and Christine Tuleau-Malot[1]

[1]Univ. Nice Sophia Antipolis, CNRS, LJAD, UMR 7351, 06100 Nice, France
[2]CEREMADE UMR CNRS 7534, Université Paris Dauphine, Place du Maréchal De Lattre De Tassigny, 75775 PARIS Cedex 16, France

Email: Patricia Reynaud-Bouret*- reynaudb@unice.fr; Vincent Rivoirard - rivoirard@ceremade.dauphine.fr; Franck Grammont - grammont@unice.fr; Christine Tuleau-Malot - malot@unice.fr;

*Corresponding author

1 Proof of Proposition 1 of [1]

Let us introduce

$$Z_p = \sqrt{p}\sup_x |F_p(x) - \hat{F}(x)| \quad \text{and} \quad W_p = \sqrt{p}\sup_x |F_p(x) - F(x)|$$

so that $W_p \xrightarrow[p\to\infty]{\mathcal{L}} \mathcal{K}$. But, with $U_p := Z_p - W_p$, $|U_p| \leq \sqrt{p}\, \sup_x |\hat{F}(x) - F(x)| \xrightarrow[p\to\infty]{\mathbb{P}} 0$. Therefore, by using the Slutsky Lemma,

$$Z_p = U_p + W_p \xrightarrow[p\to\infty]{\mathcal{L}} \mathcal{K}.$$

2 Proof that Test 1 is asymptotically of level α.

To prove that Test 1 is of level α, it is sufficient to prove Equation (2) of [1] with $\hat{F}(x) = (1 - e^{-\hat{\lambda}x})1_{x>0}$ and to apply Proposition 1 of [1]. But for all $x > 0$,

$$|\hat{F}(x) - F(x)| = e^{-\min(\lambda,\hat{\lambda})x}\left(1 - e^{-|\hat{\lambda}-\lambda|x}\right) \leq |\hat{\lambda} - \lambda| x e^{-\min(\lambda,\hat{\lambda})x} \leq \frac{|\hat{\lambda} - \lambda|}{\min(\hat{\lambda}, \lambda)} e^{-1}.$$

Therefore

$$\sqrt{p(n)}\sup_x |\hat{F}(x) - F(x)| \leq \frac{\sqrt{p(n)}|\hat{\lambda} - \lambda|}{\min(\lambda, \hat{\lambda})}.$$

It is well known that $\hat{\lambda}$ is the maximum likelihood estimate of λ and that $\sqrt{n}(\hat{\lambda}-\lambda)$ is asymptotically normal. Therefore $\sqrt{p(n)}|\hat{\lambda}-\lambda|$ tends in probability to 0 whereas $\min(\lambda,\hat{\lambda})$ tends to λ. Therefore Equation (2) of [1] is satisfied.

3 Proof of Proposition 2 of [1]

When we are dealing with Poisson processes, or more general counting processes, the previous asymptotic approach should be taken with care because the total number of points is random. Indeed if one observes a Poisson process $N^{a,p}$, aggregated over p trials, with constant intensity, then conditionnally to the event $\{N^{a,p}([0,T_{max}])=n_{tot}\}$, the repartition of the points is uniform. So the following test is exactly of level α.

1. Compute $F_{N^{a,p}([0,T_{max}])}$ as in Equation (3) of [1].
2. Compute $\sup_{t\in[0,1]}|F_{N^{a,p}([0,T_{max}]}(t)-t|$.
3. Reject when this last quantity exceeds the random quantity $k_{N^{a,p}([0,T_{max}]),1-\alpha}$, where $k_{n_{tot},1-\alpha}$ is the exact and non asymptotic quantile of KS, on the event $\{N^{a,p}([0,T_{max}])=n_{tot}\}$.

Therefore, one can easily state that under H_0: " The process is a homogeneous Poisson process", the following upper bounds holds,

$$\begin{aligned}
\mathbb{P}(\text{the previous test rejects } H_0) &= \sum_{n_{tot}=0}^{+\infty}\mathbb{P}(\text{the test rejects } H_0|N^{a,p}([0,T_{max}])=n_{tot})\mathbb{P}(N^{a,p}([0,T_{max}])=n_{tot}) \\
&\leq \alpha\sum_{n_{tot}=0}^{+\infty}\mathbb{P}(N^{a,p}([0,T_{max}])=n_{tot})=\alpha.
\end{aligned}$$

Now to turn this argument into an asymptotic argument and use $\sqrt{N^{a,p}([0,T_{max}]}\,\tilde{k}_{1-\alpha}$ instead of $k_{N^{a,p}([0,T_{max}],1-\alpha}$, we need a random version of the convergence of $KS_{n_{tot}}$. Actually one can prove the following lemma, which shows that the previous replacement leads indeed to a test of asymptotic level α.

Lemma 1. *If the p processes are homogeneous Poisson processes, then*

$$\sqrt{N^{a,p}([0,T_{max}])}\sup_{t\in[0,1]}|F_{N^{a,p}([0,T_{max}])}(t)-t|\xrightarrow[p\to\infty]{\mathcal{L}}\mathcal{K}.$$

Proof. Let W be a variable whose distribution is $\mathcal{K}$. We set

$$Z_p=\sqrt{N^{a,p}([0,T_{max}])}\sup_{t\in[0,1]}|F_{N^{a,p}([0,T_{max}])}(t)-t|.$$

Let f be a bounded continuous function and let us consider for any positive integer n_{min},

$$\begin{aligned} |\,\mathbb{E}[f(Z_p)] - \mathbb{E}[f(W)]| &= \left| \sum_{n_{tot}=0}^{+\infty} (\mathbb{E}[f(Z_p)|N^{a,p}([0,T_{max}]) = n_{tot}] - \mathbb{E}[f(W)])\,\mathbb{P}(N^{a,p}([0,T_{max}]) = n_{tot}) \right| \\ &\leq \sum_{n_{tot}\geq n_{min}}^{+\infty} \left| \mathbb{E}[f(Z_p)|N^{a,p}([0,T_{max}]) = n_{tot}] - \mathbb{E}[f(W)] \right| \mathbb{P}(N^{a,p}([0,T_{max}]) = n_{tot}) \\ &\quad + \; 2\|f\|_\infty \mathbb{P}(N^{a,p}([0,T_{max}]) < n_{min}). \end{aligned}$$

On the one hand, for any $\varepsilon > 0$, there exists n_{min} such that for any $n_{tot} \geq n_{min}$,

$$\left| \mathbb{E}[f(Z_p)|N^{a,p}([0,T_{max}]) = n_{tot}] - \mathbb{E}[f(W)] \right| < \varepsilon.$$

On the other hand, $N^{a,p}([0,T_{max}]) = \sum_{i=1}^{p} N_i([0,T_{max}])$ is a sum of p i.i.d. variables and therefore tends almost surely and in probability to infinity. Therefore there exists p_{min} such that for all $p > p_{min}$, $\mathbb{P}(N^{a,p}([0,T_{max}]) < n_{min}) < \varepsilon$, which implies that

$$|\,\mathbb{E}[f(Z_p)] - \mathbb{E}[f(W)]| \leq (1 + 2\|f\|_\infty)\varepsilon,$$

which proves the convergence in distribution. □

As already stated, if N is an inhomogeneous Poisson process with compensator Λ, $\mathcal{N} = \{\Lambda(T) : T \in N\}$ is a homogeneous Poisson process with intensity 1 by the time-rescaling theorem [2,3]. Assume now that we observe p i.i.d. Poisson processes N_i with compensator Λ. The previous transformation on each of the N_i leads to $\mathcal{N}_i$, the $\mathcal{N}_i$'s being p homogeneous Poisson processes of intensity 1 on $[0, \Lambda(T_{max})]$. One can therefore consider the aggregated process $\mathcal{N}^{a,p}$, to which we associate the c.d.f. $F_{\mathcal{N}^{a,p}([0,\Lambda(T_{max})])}$ as in Equation (3) of [1]. We can apply the previous lemma and we have:

$$\sqrt{\mathcal{N}^{a,p}([0,\Lambda(T_{max})])} \sup_{t\in[0,1]} |F_{\mathcal{N}^{a,p}([0,\Lambda(T_{max})])}(t) - t| \xrightarrow[p\to\infty]{\mathcal{L}} \mathcal{K}. \tag{1}$$

But, using the original aggregated process $N^{a,p}$, one can also write

$$\begin{aligned} F_{\mathcal{N}^{a,p}([0,\Lambda(T_{max})]}(t) &= \frac{1}{\mathcal{N}^{a,p}([0,\Lambda(T_{max})])} \sum_{T\in\mathcal{N}^{a,p}} \mathbf{1}_{\{T/\Lambda(T_{max})\leq t\}} \\ &= \frac{1}{N^{a,p}([0,T_{max}])} \sum_{X\in N^{a,p}} \mathbf{1}_{\{\Lambda(X)/\Lambda(T_{max})\leq t\}}. \end{aligned}$$

The function $\Lambda(.)/\Lambda(T_{max})$ is a continuous c.d.f. from $[0, T_{max}]$ to $[0, 1]$. Therefore we obtain:

$$\sqrt{N^{a,p}([0,T_{max}])} \sup_{x\in[0,T_{max}]} \left| \frac{1}{N^{a,p}([0,T_{max}])} \sum_{X\in N^{a,p}} \mathbf{1}_{\{X\leq x\}} - \frac{\Lambda(x)}{\Lambda(T_{max})} \right| \xrightarrow[p\to\infty]{\mathcal{L}} \mathcal{K}. \tag{2}$$

The end of the proof of Proposition 2 of [1] is then similar to the proof of Proposition 1 of [1].

3.0.1 Proof that Test 2 is asymptotically of level α

Here $\hat{F} = F_{N^{a,n}([0,T_{max}])}$. Hence, since

$$\sqrt{N^{a,n}([0,T_{max}])} \sup_x |F_{N^{a,n}([0,T_{max}])}(x) - F(x)| \xrightarrow[p\to\infty]{\mathcal{L}} \mathcal{K},$$

by the Slutsky's lemma, it is sufficient to prove that

$$N^{a,p(n)}([0,T_{max}])/N^{a,n}([0,T_{max}]) \xrightarrow[p\to\infty]{\mathbb{P}} 0, \tag{3}$$

and use Proposition 2 of [1] to conclude the proof. But since the numerator is equivalent to $p(n)\Lambda(T_{max})$ and the denominator to $n\Lambda(T_{max})$, by the law of large number, (3) is obvious.

4 Proof that Test 3 is asymptotically of level α

Here

$$\hat{F}(t) = \frac{\int_0^t \hat{\lambda}(u)du}{\int_0^{T_{max}} \hat{\lambda}(u)du}.$$

Then for all t one can write that

$$\begin{aligned}
\hat{F}(t) - F(t) &= \frac{\int_0^t \hat{\lambda}(u)du}{\int_0^{T_{max}} \hat{\lambda}(u)du} - \frac{\int_0^t \lambda(u)du}{\int_0^{T_{max}} \lambda(u)du} \\
&= \frac{\int_0^t \hat{\lambda}(u)du}{\int_0^{T_{max}} \hat{\lambda}(u)du} - \frac{\int_0^t \lambda(u)du}{\int_0^{T_{max}} \hat{\lambda}(u)du} + \frac{\int_0^t \lambda(u)du}{\int_0^{T_{max}} \hat{\lambda}(u)du} - \frac{\int_0^t \lambda(u)du}{\int_0^{T_{max}} \lambda(u)du} \\
&= \frac{\int_0^t [\hat{\lambda}(u) - \lambda(u)]du}{\int_0^{T_{max}} \hat{\lambda}(u)du} + \frac{\int_0^t \lambda(u)du}{\int_0^{T_{max}} \lambda(u)du} \frac{\int_0^{T_{max}} [\lambda(u) - \hat{\lambda}(u)]du}{\int_0^{T_{max}} \hat{\lambda}(u)du}.
\end{aligned}$$

Therefore

$$\sup_t |\hat{F}(t) - F(t)| \leq 2 \frac{\int_0^{T_{max}} |\hat{\lambda}(u) - \lambda(u)|du}{\int_0^{T_{max}} \hat{\lambda}(u)du}.$$

Therefore $\sqrt{p(n)} \sup_t |\hat{F}(t) - F(t)| \xrightarrow[p\to\infty]{\mathbb{P}} 0$ which ensures Equation (4) of [1]. Applying Proposition 2 of [1] concludes the proof.

5 Explicit construction of the cumulated process and its asymptotical properties

If N, as a general point process, has compensator Λ and conditional intensity λ, the time-rescaling theorem in its general form [2–4] states that $\mathcal{N} = \{X = \Lambda(T) : T \in N\}$ is an homogeneous Poisson process with intensity 1 until the time $\Lambda(T_{max})$ which is a random predictable time and therefore a stopping time. Here we observe p i.i.d. point processes N_i with intensity λ_i and compensator Λ_i. In particular, for all t, the $\Lambda_i(T_{max})$ are i.i.d. We apply the previous transformation to all the N_i's, hence generating the $\mathcal{N}_i$'s, p homogeneous Poisson

processes on $[0, X_i^{max}]$ with the random stopping time $X_i^{max} = \Lambda_i(T_{max})$. Let $(\mathcal{N}_{i,x})_{x\geq 0} = (\mathcal{N}_i([0,x]))_{x\geq 0}$ be the corresponding counting process. We cumulate the counting processes in the following way: for any $x \leq \sum_{i=1}^p X_i^{max}$, we set:

$$\mathcal{N}_x^{c,p} = \sum_{i=1}^{k_x} \mathcal{N}_{i,X_i^{max}} + \mathcal{N}^{(k_x)}_{x-\sum_{i=1}^{k_x} X_i^{max}}, \tag{4}$$

where k_x is the only index in $\{0, ..., (p-1)\}$, such that

$$\sum_{i=1}^{k_x} X_i^{max} \leq x < \sum_{i=1}^{k_x+1} X_i^{max}.$$

Because each X_i^{max} is a stopping time, due to the strong Markov property of Poisson processes, the cumulation still guarantees that the jumps of $\mathcal{N}^{c,p}$, that are identified with the points of the point process $\mathcal{N}^{c,p}$, form an homogeneous Poisson process of intensity 1 on $[0, \sum_{i=1}^p X_i^{max}]$. Let us fix some $\theta > 0$ such that $\mathbb{E}[\Lambda_i(T_{max})] > \theta$. One can prove the following result.

Lemma 2. *For all $\theta > 0$ such that $\mathbb{E}(\Lambda_i(T_{max})) > \theta$,*

$$\sqrt{\mathcal{N}^{c,p}([0,p\theta])} \sup_{u\in[0,1]} \left| \frac{1}{\mathcal{N}^{c,p}([0,p\theta])} \sum_{X\in\mathcal{N}^{c,p}, X\leq p\theta} \mathbf{1}_{\{X/(p\theta)\leq u\}} - u \right| \xrightarrow[p\to\infty]{\mathcal{L}} \mathcal{K}.$$

Proof. Using the cumulation described in (4), let us complete $\mathcal{N}^{c,p}$ with another independent homogeneous Poisson process with intensity 1 and infinite support beyond $\sum_{i=1}^p X_i^{max}$, hence obtaining $\mathcal{N}'$ an homogeneous Poisson process of intensity 1 on $\mathbb{R}_+$. Let us denote

$$Z_p = \sqrt{\mathcal{N}^{c,p}([0,p\theta])} \sup_{u\in[0,1]} \left| \frac{1}{\mathcal{N}^{c,p}([0,p\theta])} \sum_{X\in\mathcal{N}^{c,p}, X\leq p\theta} \mathbf{1}_{\{X/(p\theta)\leq u\}} - u \right|.$$

We define Z'_p with the same expression except that $\mathcal{N}^{c,p}$ is replaced by $\mathcal{N}'$. The point process defined by $\{X/(p\theta)/X \in \mathcal{N}' \cap [0,p\theta]\}$ is a Poisson process with intensity $p\theta$ on $[0,1]$. It can therefore also be viewed as an aggregated process of p i.i.d Poisson processes with intensity θ. Therefore Z'_p can be viewed as the quantity appearing in Lemma 1 showing that Z'_p tends in distribution to $\mathcal{K}$. So following the same proof, it remains to show that for any bounded continuous function f, $|\mathbb{E}(f(Z_p)) - \mathbb{E}(f(Z'_p))|$ tends to 0. But $\mathcal{N}^{c,p} \cap [0,p\theta] = \mathcal{N}' \cap [0,p\theta]$ on the event $\{\sum_{i=1}^p X_i^{max} > p\theta\}$. Therefore $|\mathbb{E}(f(Z_p)) - \mathbb{E}(f(Z'_p))| \leq 2\|f\|_\infty \mathbb{P}(\sum_{i=1}^p X_i^{max} \leq p\theta)$. But by the law of large numbers,

$$\frac{1}{p}\sum_{i=1}^p X_i^{max} \xrightarrow[p\to\infty]{\mathbb{P}} \mathbb{E}(\Lambda_i(T_{max})) > \theta.$$

Hence $\mathbb{P}(\sum_{i=1}^p X_i^{max} \leq p\theta)$ tends to 0, which concludes the proof. ☐

One can go back to the classical time t by introducing the cumulated point process

$$\forall t \geq 0, \quad N_t^{c,p} = \sum_{i=1}^{j_t} N_{i,T_{max}} + N_{j_t, t-j_t T_{max}},$$

where $j_t = \lfloor t/T_{max} \rfloor$. One can also introduce

$$\forall t \ \geq 0, \quad \Lambda^{c,p}(t) = \sum_{i=1}^{j_t} \Lambda_i(T_{max}) + \Lambda^{(j_t)}(t - j_t T_{max}).$$

The function $\Lambda^{c,p}(.)$ is a continuous non decreasing function and therefore, one can consider its generalized inverse function $(\Lambda^{c,p})^{-1}$. Therefore one can rewrite Lemma 2 as follows:

$$\sqrt{N^{c,p}([0,(\Lambda^{c,p})^{-1}(p\theta)])} \sup_{t \in [0,(\Lambda^{c,p})^{-1}(p\theta))]} \left| \frac{1}{N^{c,p}([0,(\Lambda^{c,p})^{-1}(p\theta)])} \sum_{T \in N^{c,p}, T \leq (\Lambda^{c,p})^{-1}(p\theta)} \mathbf{1}_{\{T \leq t\}} - \frac{\Lambda^{c,p}(t)}{p\theta} \right| \xrightarrow[p\to\infty]{\mathcal{L}} \mathcal{K}.$$

6 Proof of Theorem 1 of [1]

Now we want to replace $\Lambda^{c,p}$ by an estimate of the type

$$\forall\, t \geq 0, \quad \hat{\Lambda}^{c,p}(t) = \sum_{i=1}^{j_t} \int_0^{T_{max}} \hat{\lambda}_i du + \int_0^{t - j_t T_{max}} \hat{\lambda}^{(j_t)} du.$$

Since $\hat{\Lambda}^{c,p}$ is also continuous and non-decreasing, one has the following equality:

$$\sup_{t \in [0,(\hat{\Lambda}^{c,p})^{-1}(p\theta)]} \left| \frac{1}{N^{c,p}((\hat{\Lambda}^{c,p})^{-1}(p\theta))} \sum_{T \in N^{c,p}, T \leq (\hat{\Lambda}^{c,p})^{-1}(p\theta)} \mathbf{1}_{\{T \leq t\}} - \frac{\hat{\Lambda}^{c,p}(t)}{p\theta} \right| =$$

$$\sup_{u \in [0,1]} \left| \frac{1}{\hat{\mathcal{N}}^{c,p}([0,p\theta])} \sum_{X \in \hat{\mathcal{N}}^{c,p}, X \leq p\theta} \mathbf{1}_{\{X/(p\theta) \leq u\}} - u \right|,$$

with $\hat{\mathcal{N}}^{c,p}$ the cumulated process obtained with $\hat{\mathcal{N}}_i = \{X = \int_0^T \hat{\lambda}_i(u)du : T \in N_i\}$ instead of $\mathcal{N}_i$ in (4).

Since $\Lambda^{c,p}(pT_{max})/p = \sum_i X_i^{max}/p$ tends in probability to $\mathbb{E}(\Lambda_i(T_{max})) > \theta$, with probability tending to 1, $p\theta$ belongs to $[0, \Lambda^{c,p}(pT_{max})]$. Moreover,

$$\left| \frac{\hat{\Lambda}^{c,p}(pT_{max}) - \Lambda^{c,p}(pT_{max})}{p} \right| \leq \frac{1}{p} \sum_{i=1}^{p} \int_0^{T_{max}} \left| \hat{\lambda}_i(u) - \lambda_i(u) \right| du,$$

where the right hand side tends to 0 in probability. Therefore $\hat{\Lambda}^{c,p}(pT_{max})/p$ also tends in probability to $\mathbb{E}(\Lambda_i(T_{max})) > \theta$ and with probability tending to 1, $p\theta$ belongs to $[0, \hat{\Lambda}^{c,p}(pT_{max})]$. Therefore both $(\hat{\Lambda}^{c,p})^{-1}(p\theta)$ and $(\Lambda^{c,p})^{-1}(p\theta)$ are strictly smaller than pT_{max} with probability tending to 1. Furthermore,

if $(\hat{\Lambda}^{c,p})^{-1}(p\theta) < pT_{max}$ and $(\Lambda^{c,p})^{-1}(p\theta) < pT_{max}$,

$$\begin{aligned}\left|\Lambda^{c,p}((\hat{\Lambda}^{c,p})^{-1}(p\theta)) - \Lambda^{c,p}((\Lambda^{c,p})^{-1}(p\theta))\right| &= \left|\Lambda^{c,p}((\hat{\Lambda}^{c,p})^{-1}(p\theta)) - p\theta\right| \\ &= \left|\Lambda^{c,p}((\hat{\Lambda}^{c,p})^{-1}(p\theta)) - \hat{\Lambda}^{c,p}((\hat{\Lambda}^{c,p})^{-1}(p\theta))\right| \\ &\leq \sum_{i=1}^{p} \int_0^{T_{max}} \left|\hat{\lambda}_i(u) - \lambda_i(u)\right| du.\end{aligned}$$

Hence, by assumption, for $\delta > 0$, if

$$\Omega_\delta = \left\{\left|\Lambda^{c,p}((\hat{\Lambda}^{c,p})^{-1}(p\theta)) - \Lambda^{c,p}((\Lambda^{c,p})^{-1}(p\theta))\right| \leq \delta\sqrt{p}\right\},$$

for any $\varepsilon > 0$, there exists p_0, such that for any $p \geq p_0$, $\mathbb{P}(\Omega_\delta) \geq 1 - \epsilon$. On Ω_δ, one has therefore that

$$p\theta - \delta\sqrt{p} \leq \Lambda^{c,p}((\hat{\Lambda}^{c,p})^{-1}(p\theta)) \leq p\theta + \delta\sqrt{p}$$

and

$$\begin{aligned}\left|N^{c,p}([0,(\hat{\Lambda}^{c,p})^{-1}(p\theta)]) - N^{c,p}([0,(\Lambda^{c,p})^{-1}(p\theta)])\right| &= \left|\mathcal{N}^{c,p}([0,\Lambda^{c,p}((\hat{\Lambda}^{c,p})^{-1}(p\theta))]) - \mathcal{N}^{c,p}([0,p\theta])\right| \\ &\leq \max\left[\mathcal{N}^{c,p}([0,p\theta+\delta\sqrt{p}]) - \mathcal{N}^{c,p}([0,p\theta])\ ,\ \mathcal{N}^{c,p}([0,p\theta]) - \mathcal{N}^{c,p}([0,p\theta-\delta\sqrt{p}])\right].\end{aligned}$$

In the previous expression, we consider the maximum of two independent Poisson variables (denoted U and V) with parameter $\delta\sqrt{p}$. For any $u > 0$,

$$\begin{aligned}\mathbb{P}\left[\left|N^{c,p}([0,(\hat{\Lambda}^{c})^{-1}(p\theta)]) - N^{c,p}([0,(\Lambda^{c})^{-1}(p\theta)])\right| \geq (\delta+u)\sqrt{p} \text{ or } \Omega_\delta^c\right] &\leq \epsilon + \mathbb{P}(\max\{U,V\} \geq (\delta+u)\sqrt{p}) \\ &\leq \epsilon + 2\exp\left(-\frac{pu^2}{2\sqrt{p}\delta + \sqrt{p}u}\right).\end{aligned}$$

By taking $u = \delta$, the last expression shows that

$$p^{-1/2}|N^{c,p}([0,(\hat{\Lambda}^{c,p})^{-1}(p\theta)]) - N^{c,p}([0,(\Lambda^{c,p})^{-1}(p\theta)])| \xrightarrow[p\to\infty]{\mathbb{P}} 0. \tag{5}$$

If we are able to show that

$$\sqrt{\mathcal{N}^{c,p}([0,p\theta])}\sup_{u\in[0,1]}\left|\frac{1}{\mathcal{N}^{c,p}([0,p\theta])}\sum_{X\in\mathcal{N}^{c,p},X\leq p\theta}\mathbf{1}_{\{X/(p\theta)\leq u\}} - u\right| \xrightarrow[p\to\infty]{\mathcal{L}} \mathcal{K},$$

since $N^{c,p}([0,(\hat{\Lambda}^{c,p})^{-1}(p\theta)])/N^{c,p}([0,(\Lambda^{c,p})^{-1}(p\theta)])$ tends to 1 in probability, this will imply the result by using Slustky's Lemma. Now, we clip $\Lambda^{c,p}$ and $\hat{\Lambda}^{c,p}$ and we set for any t,

$$\bar{\Lambda}^{c,p}(t) = \min(\Lambda^{c,p}(t), p\theta) \quad \text{and} \quad \bar{\hat{\Lambda}}^{c,p}(t) = \min(\hat{\Lambda}^{c,p}(t), p\theta).$$

Therefore, $\bar{\Lambda}^{c,p}(\cdot)/(p\theta)$ and $\bar{\hat{\Lambda}}^{c,p}(\cdot)/(p\theta)$ are continuous c.d.f. and

$$\sup_{u\in[0,1]}\left|\frac{1}{\hat{\mathcal{N}}^{c,p}([0,p\theta])}\sum_{X\in\hat{\mathcal{N}}^{c,p},X\leq p\theta}\mathbf{1}_{\{X/(p\theta)\leq u\}}-u\right|=\sup_{t>0}\left|\frac{1}{N^{c,p}([0,(\bar{\hat{\Lambda}}^{c,p})^{-1}(p\theta)])}\sum_{T\in N^{c,p},T\leq(\bar{\hat{\Lambda}}^{c,p})^{-1}(p\theta)}\mathbf{1}_{\{T\leq t\}}-\frac{\bar{\hat{\Lambda}}^{c,p}(t)}{p\theta}\right|.$$

But since $p\theta$ belongs to $[0,\hat{\Lambda}^{c,p}(pT_{max})]$ with probability tending to 1, the right hand side is equal to

$$A_p=\sup_{t>0}\left|\frac{1}{N^{c,p}([0,(\hat{\Lambda}^{c,p})^{-1}(p\theta)])}\sum_{T\in N^{c,p},T\leq(\hat{\Lambda}^{c,p})^{-1}(p\theta)}\mathbf{1}_{\{T\leq t\}}-\frac{\bar{\hat{\Lambda}}^{c,p}(t)}{p\theta}\right|$$

with probability tending to 1. So it is sufficient to prove that $\hat{Z}_p:=\sqrt{\mathcal{N}^{c,p}([0,p\theta])}A_p$ tends in distribution to $\mathcal{K}$. Note that

$$\hat{Z}_p=\sqrt{N^{c,p}([0,(\Lambda^{c,p})^{-1}(p\theta)])}\sup_{t>0}\left|\frac{1}{N^{c,p}([0,(\hat{\Lambda}^{c,p})^{-1}(p\theta)])}\sum_{T\in N^{c,p},T\leq(\hat{\Lambda}^{c,p})^{-1}(p\theta)}\mathbf{1}_{\{T\leq t\}}-\frac{\bar{\hat{\Lambda}}^{c,p}(t)}{p\theta}\right|.$$

We denote:

$$\begin{aligned}\tilde{Z}_p&=\sqrt{N^{c,p}([0,(\Lambda^{c,p})^{-1}(p\theta)])}\sup_{t>0}\left|\frac{1}{N^{c,p}([0,(\Lambda^{c,p})^{-1}(p\theta)])}\sum_{T\in N^{c,p},T\leq(\Lambda^{c,p})^{-1}(p\theta)}\mathbf{1}_{\{T\leq t\}}-\frac{\bar{\hat{\Lambda}}^{c,p}(t)}{p\theta}\right|\\ Z_p&=\sqrt{N^{c,p}([0,(\Lambda^{c,p})^{-1}(p\theta)])}\sup_{t>0}\left|\frac{1}{N^{c,p}([0,(\Lambda^{c,p})^{-1}(p\theta)])}\sum_{T\in N^{c,p},T\leq(\Lambda^{c,p})^{-1}(p\theta)}\mathbf{1}_{\{T\leq t\}}-\frac{\bar{\Lambda}^{c,p}(t)}{p\theta}\right|.\end{aligned}$$

But Z_p is also equal with probability tending to 1 to

$$\sqrt{N^{c,p}([0,(\Lambda^{c,p})^{-1}(p\theta)])}\sup_{t>0}\left|\frac{1}{N^{c,p}([0,(\bar{\Lambda}^{c,p})^{-1}(p\theta)])}\sum_{T\in N^{c,p},T\leq(\bar{\Lambda}^{c,p})^{-1}(p\theta)}\mathbf{1}_{\{T\leq t\}}-\frac{\bar{\Lambda}^{c,p}(t)}{p\theta}\right|=$$
$$\sqrt{N^{c,p}([0,(\Lambda^{c,p})^{-1}(p\theta)])}\sup_{u\in[0,1]}\left|\frac{1}{\mathcal{N}^{c,p}([0,p\theta])}\sum_{X\in\mathcal{N}^{c,P},X\leq p\theta}\mathbf{1}_{\{X/(p\theta)\leq u\}}-u\right|,$$

because $p\theta$ belongs to $[0,\Lambda^{c,p}(pT_{max})]$ with probability tending to 1. Using Lemma 2 as before, we have that Z_p tends in distribution to $\mathcal{K}$. It is consequently sufficient to prove that $\hat{Z}_p-\tilde{Z}_p$ and $\tilde{Z}_p-Z_p$ tend both in probability to 0. We have:

$$\begin{aligned}\left|\hat{Z}_p-\tilde{Z}_p\right|&\leq\sqrt{N^{c,p}([0,(\Lambda^{c,p})^{-1}(p\theta)])}\sup_{t>0}\left|\frac{1}{N^{c,p}([0,(\Lambda^{c,p})^{-1}(p\theta)])}\sum_{T\in N^{c,p},T\leq(\Lambda^{c,p})^{-1}(p\theta)}\mathbf{1}_{\{T\leq t\}}\right.\\&\qquad\left.-\frac{1}{N^{c,p}([0,(\hat{\Lambda}^{c,p})^{-1}(p\theta)])}\sum_{T\in N^{c,p},T\leq(\hat{\Lambda}^{c,p})^{-1}(p\theta)}\mathbf{1}_{\{T\leq t\}}\right|\\&\leq 2\sqrt{\mathcal{N}^{c,p}([0,p\theta])}\frac{\left|N^{c,p}([0,(\hat{\Lambda}^{c,p})^{-1}(p\theta)])-N^{c,p}([0,(\Lambda^{c,p})^{-1}(p\theta)])\right|}{\max\left(N^{c,p}([0,(\hat{\Lambda}^{c,p})^{-1}(p\theta)]),N^{c,p}([0,(\Lambda^{c,p})^{-1}(p\theta)])\right)},\end{aligned}$$

which tends to 0 in probability by (5). Furthermore, if $(\hat{\Lambda}^{c,p})^{-1}(p\theta) < pT_{max}$ and $(\Lambda^{c,p})^{-1}(p\theta) < pT_{max}$,

$$\begin{aligned}
\left|\tilde{Z}_p - Z_p\right| &\leq \sqrt{N^{c,p}([0,(\Lambda^{c,p})^{-1}(p\theta)])}\sup_{t>0}\left|\frac{\bar{\hat{\Lambda}}^{c,p}(t)}{p\theta} - \frac{\bar{\Lambda}^{c,p}(t)}{p\theta}\right| \\
&\leq \frac{\sqrt{N^{c,p}([0,(\Lambda^{c,p})^{-1}(p\theta)])}}{p\theta}\sup_{t\leq pT_{max}}\left|\hat{\Lambda}^{c,p}(t) - \Lambda^{c,p}(t)\right| \\
&\leq \frac{\sqrt{\mathcal{N}^{c,p}([0,p\theta])}}{p\theta}\sum_{i=1}^{p}\int_0^{T_{max}}\left|\hat{\lambda}_i(u) - \lambda_i(u)\right| du.
\end{aligned}$$

Since $\mathcal{N}^{c,p}([0,p\theta])$ is also the sum of p i.i.d. Poisson variables, it behaves like a multiple of p by the law of large numbers. Therefore, by using Equation (7) of [1], $\tilde{Z}_p - Z_p \xrightarrow[p\to\infty]{\mathbb{P}} 0$.

7 Proof that Test 4 is of level α asymptotically

We apply Theorem 1 of [1] with $\hat{\lambda}_i = ((\lambda_i)_{\hat{f}})_+$. Since the λ_i's are positive, one has that for all u,

$$\left|\hat{\lambda}_i(u) - \lambda_i(u)\right| \leq \left|(\lambda_i)_{\hat{f}}(u) - \lambda_i(u)\right|,$$

which gives exactly Equation (7) of [1].

References

1. Reynaud-Bouret P, Rivoirard V, Grammont F, Tuleau-Malot C: **Goodness-of-fit tests and nonparametric adaptive estimation for spike train analysis**. Tech. rep., http://hal.archives-ouvertes.fr/hal-00789127 2013.
2. Daley DJ, Vere-Jones D: *An introduction to the theory of point processes. Vol. I.* Probability and its Applications (New York), New York: Springer-Verlag, second edition 2003. [Elementary theory and methods].
3. Brown E, Barbieri R, Ventura V, Kass R, Frank L: **The time rescaling theorem and its application to neural spike train analysis.** *Neural Computation* 2002, **14**(2):325–346.
4. Brémaud P: *Point processes and queues.* New York: Springer-Verlag 1981. [Martingale dynamics, Springer Series in Statistics].
