# Supplementary material for: Goodness-of-Fit Tests and Nonparametric Adaptive Estimation for Spike Train Analysis
Source: J Math Neurosci. 2014 Apr 17;4:3. doi: 10.1186/2190-8567-4-3 (PMC4021619; doi:10.1186/2190-8567-4-3)
Supplement: Additional file 2 — Proof of Theorem 2 (PDF 94.6 KB) [file 2190-8567-4-3-S2.pdf]

Additional File 2 of "Goodness-of-fit tests and nonparametric adaptive estimation for spike train analysis": proof of Theorem 2 of [1]

Patricia Reynaud-Bouret*[1] and Vincent Rivoirard[2] and Franck Grammont[1] and Christine Tuleau-Malot[1]

[1]Univ. Nice Sophia Antipolis, CNRS, LJAD, UMR 7351, 06100 Nice, France
[2]CEREMADE UMR CNRS 7534, Université Paris Dauphine, Place du Maréchal De Lattre De Tassigny, 75775 PARIS Cedex 16, France

Email: Patricia Reynaud-Bouret*- reynaudb@unice.fr; Vincent Rivoirard - rivoirard@ceremade.dauphine.fr; Franck Grammont - grammont@unice.fr; Christine Tuleau-Malot - malot@unice.fr;

*Corresponding author

In this additional file, the intensity $\lambda(.)$ should be understood as a function on the whole real line which is null outside $[0, T_{max}]$. In particular, the Poisson process $N^{a,n}$, with intensity $n\lambda(.)$, exists now on $\mathbb{R}$, but there is no points outside $[0, T_{max}]$ and $N^{a,n}(\mathbb{R}) = N^{a,n}([0, T_{max}])$. The proof is inspired by Proposition 2 of [2]. We set:

$$\chi = (1+\eta)(1+\|K\|_1)\|K\|_2.$$

Therefore, we have:

$$A(h) = \sup_{h' \in \mathcal{H}} \left\{ \|\hat{\lambda}_n^{h,h'} - \hat{\lambda}_n^{K_{h'}}\|_2 - \frac{\chi\sqrt{N^{a,n}(\mathbb{R})}}{n\sqrt{h'}} \right\}_+$$

and

$$\hat{h} = \arg\min_{h \in \mathcal{H}} \left\{ A(h) + \frac{\chi\sqrt{N^{a,n}(\mathbb{R})}}{n\sqrt{h}} \right\}.$$

For any $h \in \mathcal{H}$,

$$\|\hat{\lambda}_n^{GL} - \lambda\|_2 \le A_1 + A_2 + A_3,$$

with

$$A_1 := \|\hat{\lambda}_n^{GL} - \hat{\lambda}_n^{\hat{h},h}\|_2 \le A(h) + \frac{\chi\sqrt{N^{a,n}(\mathbb{R})}}{n\sqrt{\hat{h}}},$$

$$A_2 := \|\hat{\lambda}_n^{\hat{h},h} - \hat{\lambda}_n^{K_h}\|_2 \le A(\hat{h}) + \frac{\chi\sqrt{N^{a,n}(\mathbb{R})}}{n\sqrt{h}}$$

and

$$A_3 := \|\hat{\lambda}_n^{K_h} - \lambda\|_2.$$

By definition of $\hat{h}$, we have:

$$A_1 + A_2 \le 2A(h) + \frac{2\chi\sqrt{N^{a,n}(\mathbb{R})}}{n\sqrt{h}}.$$

Therefore, by setting

$$\zeta_n(h) := \sup_{h'\in\mathcal{H}} \left\{ \|(\hat{\lambda}_n^{h,h'} - \mathbb{E}[\hat{\lambda}_n^{h,h'}]) - (\hat{\lambda}_n^{K_{h'}} - \mathbb{E}[\hat{\lambda}_n^{K_{h'}}])\|_2 - \frac{\chi\sqrt{N^{a,n}(\mathbb{R})}}{n\sqrt{h'}} \right\}_+$$

we have:

$$\begin{aligned}
A_1 + A_2 &\le 2\zeta_n(h) + 2\sup_{h'\in\mathcal{H}} \|\,\mathbb{E}[\hat{\lambda}_n^{h,h'}] - \mathbb{E}[\hat{\lambda}_n^{K_{h'}}]\|_2 + \frac{2\chi\sqrt{N^{a,n}(\mathbb{R})}}{n\sqrt{h}} \\
&\le 2\zeta_n(h) + 2\sup_{h'\in\mathcal{H}} \|K_h \star K_{h'} \star \lambda - K_{h'} \star \lambda\|_2 + \frac{2\chi\sqrt{N^{a,n}(\mathbb{R})}}{n\sqrt{h}} \\
&\le 2\zeta_n(h) + 2\|K\|_1\|K_h \star \lambda - \lambda\|_2 + \frac{2\chi\sqrt{N^{a,n}(\mathbb{R})}}{n\sqrt{h}}.
\end{aligned}$$

Finally, since $(a+b+c)^2 \le 3a^2 + 3b^2 + 3c^2$,

$$\begin{aligned}
\mathbb{E}[(A_1+A_2)^2] &\le 12\,\mathbb{E}[\zeta_n^2(h)] + 12\|K\|_1^2\|K_h \star \lambda - \lambda\|_2^2 + \frac{12\chi^2\,\mathbb{E}[N^{a,n}(\mathbb{R})]}{n^2 h} \\
&\le 12\,\mathbb{E}[\zeta_n^2(h)] + 12\|K\|_1^2\|K_h \star \lambda - \lambda\|_2^2 + \frac{12\chi^2\|\lambda\|_1}{nh}.
\end{aligned}$$

For the last term, we obtain:

$$\begin{aligned}
\mathbb{E}[A_3^2] &= \int \operatorname{Var}(\hat{\lambda}_n^{K_h}(x))dx + \|K_h \star \lambda - \lambda\|_2^2 \\
&= \frac{1}{n^2}\iint K_h^2(x-u)n\lambda(u)dudx + \|K_h \star \lambda - \lambda\|_2^2 \\
&= \frac{\|\lambda\|_1}{nh}\|K\|_2^2 + \|K_h \star \lambda - \lambda\|_2^2.
\end{aligned}$$

Finally, replacing χ with its definition, we obtain: for any $h \in \mathcal{H}$,

$$\begin{aligned}
\mathbb{E}\,\|\hat{\lambda}_n^{GL} - \lambda\|_2^2 &\le 2\,\mathbb{E}[(A_1+A_2)^2] + 2\,\mathbb{E}[A_3^2] \\
&\le 2(1+12\|K\|_1^2)\|K_h \star \lambda - \lambda\|_2^2 + 2(1+12(1+\eta)^2(1+\|K\|_1)^2)\frac{\|\lambda\|_1}{nh}\|K\|_2^2 + 24\,\mathbb{E}[\zeta_n^2(h)].
\end{aligned}$$

The result follows by using the next lemma.

Lemma 1. *If $\mathcal{H} \subset \{D^{-1} :\ D = 1,...,D_{\max}\}$ with $D_{\max} = \delta n$ for some $\delta > 0$, and if $\|\lambda\|_\infty < \infty$, then there exists a constant C depending on δ, η, $\|K\|_2$, $\|K\|_1$, $\|\lambda\|_1$ and $\|\lambda\|_\infty$ such that for any $h \in \mathcal{H}$*

$$\mathbb{E}[\zeta_n^2(h)] \le Cn^{-1}.$$

Proof. First, we notice that for any $h \in \mathcal{H}$

$$\begin{aligned}
\zeta_n(h) &\leq \sup_{h'\in\mathcal{H}} \left\{ \|\hat{\lambda}_n^{h,h'} - \mathbb{E}[\hat{\lambda}_n^{h,h'}]\|_2 + \|\hat{\lambda}_n^{K_{h'}} - \mathbb{E}[\hat{\lambda}_n^{K_{h'}}]\|_2 - \frac{\chi\sqrt{N^{a,n}(\mathbb{R})}}{n\sqrt{h'}} \right\}_+ \\
&\leq \sup_{h'\in\mathcal{H}} \left\{ (\|K\|_1+1)\|\hat{\lambda}_n^{K_{h'}} - \mathbb{E}[\hat{\lambda}_n^{K_{h'}}]\|_2 - \frac{\chi\sqrt{N^{a,n}(\mathbb{R})}}{n\sqrt{h'}} \right\}_+ \\
&\leq (\|K\|_1+1)S_n,
\end{aligned}$$

with

$$S_n := \sup_{h\in\mathcal{H}} \left\{ \|\hat{\lambda}_n^{K_h} - \mathbb{E}[\hat{\lambda}_n^{K_h}]\|_2 - \frac{\|K\|_2(1+\eta)\sqrt{N^{a,n}(\mathbb{R})}}{n\sqrt{h}} \right\}_+ .$$

We then have:

$$\mathbb{E}[\zeta_n^2(h)] \leq (\|K\|_1+1)^2(A+B)$$

with

$$A := \mathbb{E}[S_n^2 1_{\{N^{a,n}(\mathbb{R})\leq(1-\alpha)^2 n\|\lambda\|_1\}}],$$

$$B := \mathbb{E}[S_n^2 1_{\{N^{a,n}(\mathbb{R})>(1-\alpha)^2 n\|\lambda\|_1\}}]$$

for all $\alpha \in (0,1)$. We have:

$$\begin{aligned}
S_n^2 &\leq 2\sup_{h\in\mathcal{H}} \|\hat{\lambda}_n^{K_h}\|_2^2 + 2\sup_{h\in\mathcal{H}} \|\,\mathbb{E}[\hat{\lambda}_n^{K_h}]\|_2^2 \\
&\leq 2n^{-2}\sup_{h\in\mathcal{H}} \int dx \left(\int K_h(x-u)dN^{a,n}(u) \right)^2 + 2\sup_{h\in\mathcal{H}} \|K_h \star \lambda\|_2^2 \\
&\leq 2n^{-2}\sup_{h\in\mathcal{H}} \iint K_h^2(x-u)dN^{a,n}(u))dx \times N^{a,n}(\mathbb{R}) + 2\sup_{h\in\mathcal{H}} \|K_h \star \lambda\|_2^2 \\
&\leq 2n^{-2}\sup_{h\in\mathcal{H}} \frac{\|K\|_2^2}{h} \times N^{a,n}(\mathbb{R})^2 + 2\sup_{h\in\mathcal{H}} \frac{\|K\|_2^2}{h}\|\lambda\|_1^2 \\
&\leq 2\delta n\|K\|_2^2 \left(\frac{N^{a,n}(\mathbb{R})^2}{n^2} + \|\lambda\|_1^2 \right).
\end{aligned}$$

Therefore,

$$A \leq 4\delta n\|K\|_2^2\|\lambda\|_1^2 \times \mathbb{P}(N^{a,n}(\mathbb{R}) \leq (1-\alpha)^2 n\|\lambda\|_1).$$

To bound the last term, we use, for instance, Inequality (5.2) of [3] (with $\xi = (2\alpha-\alpha^2)n\|\lambda\|_1$ and with the function $f \equiv -1$), which shows that there exists $\alpha' > 0$ only depending on α such that

$$\mathbb{P}(N^{a,n}(\mathbb{R}) \leq (1-\alpha)^2 n\|\lambda\|_1) \leq \exp(-\alpha'\|\lambda\|_1 \times n).$$

This shows that

$$A \leq C_A n^{-1},$$

where C_A depends on α, δ, $\|K\|_2$ and $\|\lambda\|_1$. Now, we deal with the term B by fixing the previous value α: we set $\alpha = \min(\eta/2, 1/4)$. This implies

$$(1+\eta)(1-\alpha) \geq 1 + \frac{\eta}{4}$$

and

$$\begin{aligned}
B &= \mathbb{E}[S_n^2 1_{\{N^{a,n}(\mathbb{R})>(1-\alpha)^2 n\|\lambda\|_1\}}] \\
&\leq \mathbb{E}\left[\sup_{h\in\mathcal{H}}\left\{\|\hat{\lambda}_n^{K_h} - \mathbb{E}[\hat{\lambda}_n^{K_h}]\|_2 - \frac{(1+\frac{\eta}{4})\|K\|_2\sqrt{\|\lambda\|_1}}{\sqrt{nh}}\right\}_+^2\right] \\
&= \int_0^{+\infty} \mathbb{P}\left(\sup_{h\in\mathcal{H}}\left\{\|\hat{\lambda}_n^{K_h} - \mathbb{E}[\hat{\lambda}_n^{K_h}]\|_2 - \frac{(1+\frac{\eta}{4})\|K\|_2\sqrt{\|\lambda\|_1}}{\sqrt{nh}}\right\}_+^2 \geq x\right) dx \\
&\leq \sum_{h\in\mathcal{H}} \int_0^{+\infty} \mathbb{P}\left(\left\{\|\hat{\lambda}_n^{K_h} - \mathbb{E}[\hat{\lambda}_n^{K_h}]\|_2 - \frac{(1+\frac{\eta}{4})\|K\|_2\sqrt{\|\lambda\|_1}}{\sqrt{nh}}\right\}_+^2 \geq x\right) dx.
\end{aligned}$$

To conclude, it remains to control for any $x \geq 0$, the probability inside the integral. For this purpose, we apply Corollary 2 of [3] and we set

$$U(x) = \hat{\lambda}_n^{K_h}(x) - \mathbb{E}[\hat{\lambda}_n^{K_h}(x)] = \frac{1}{n}\int K_h(x-u)dN^{a,n}(u) - (K_h \star \lambda)(x).$$

If $\mathcal{A}$ is a countable dense subset of the unit ball of $\mathbb{L}_2(\mathbb{R})$, we have:

$$\begin{aligned}
\|U\|_2 &= \sup_{a\in\mathcal{A}} \int a(t)U(t)dt \\
&= \sup_{a\in\mathcal{A}} \int a(t)\left(\frac{1}{n}\int K_h(t-u)dN^{a,n}(u) - \int K_h(t-u)\lambda(u)du\right)dt \\
&= \sup_{a\in\mathcal{A}} \int \psi_a(u)(dN^{a,n}(u) - n\lambda(u))du,
\end{aligned}$$

with for any $a \in \mathcal{A}$ and any $u \in \mathbb{R}$,

$$\psi_a(u) = \frac{1}{n}\int a(t)K_h(t-u)dt.$$

We have for any u,

$$\psi_a^2(u) \leq \frac{1}{n^2}\int a^2(t)dt \int K_h^2(t-u)dt = \frac{\|K\|_2^2}{n^2 h}.$$

So, if $b = \frac{\|K\|_2}{n\sqrt{h}}$, $\|\psi_a\|_\infty \leq b$. We have

$$\mathbb{E}[\|U\|_2^2] = \int \mathbb{E}[U^2(t)]dt = \int \text{var}(\hat{\lambda}_n^{K_h}(t))dt = \frac{\|\lambda\|_1}{nh}\|K\|_2^2,$$

which implies

$$\mathbb{E}[\|U\|_2] \le \frac{\sqrt{\|\lambda\|_1}}{\sqrt{nh}}\|K\|_2.$$

Finally,

$$\begin{aligned} v &:= \sup_{a\in\mathcal{A}} \int \psi_a^2(x) n\lambda(x)dx \\ &= \sup_{a\in\mathcal{A}} \int \left(\int \frac{a(t)}{n} K_h(t-x)dt\right)^2 n\lambda(x)dx \\ &\le \frac{1}{n}\int \lambda(x)dx \int a^2(t)|K_h(t-x)|dt \int |K_h(t-x)|dt \\ &\le \frac{\|K\|_1^2\|\lambda\|_\infty}{n}. \end{aligned}$$

Corollary 2 of [3] gives: for any $\epsilon > 0$, for any $u > 0$,

$$\mathbb{P}\left(\|\hat{\lambda}_n^{K_h} - \mathbb{E}[\hat{\lambda}_n^{K_h}]\|_2 \ge (1+\epsilon)\frac{\sqrt{\|\lambda\|_1}}{\sqrt{nh}}\|K\|_2 + \sqrt{\frac{12}{n}\|K\|_1^2\|\lambda\|_\infty u} + \left(\frac{5}{4}+\frac{32}{\epsilon}\right)\frac{\|K\|_2}{n\sqrt{h}}u\right) \le \exp(-u).$$

Then, we conclude by using exactly the same computation as in the proof of Lemma 1 of [4, p 32-34] that gives:

$$B \le C_B n^{-1},$$

where C_B is a constant depending on δ, η, $\|K\|_2$, $\|K\|_1$ and $\|\lambda\|_\infty$. □

The lemma leads to the result.

References

1. Reynaud-Bouret P, Rivoirard V, Grammont F, Tuleau-Malot C: **Goodness-of-fit tests and nonparametric adaptive estimation for spike train analysis**. Tech. rep., http://hal.archives-ouvertes.fr/hal-00789127 2013.
2. Doumic M, Hoffmann M, Reynaud-Bouret P, Rivoirard V: **Nonparametric estimation of the division rate of a size-structured population**. *SIAM J. Numer. Anal.* 2012, **50**(2):925–950.
3. Reynaud-Bouret P: **Adaptive estimation of the intensity of inhomogeneous Poisson processes via concentration inequalities**. *Probab. Theory Related Fields* 2003, **126**:103–153.
4. Doumic M, Hoffmann M, Reynaud-Bouret P, Rivoirard V: **Nonparametric estimation of the division rate of a size-structured population**. Tech. rep., http://hal.archives-ouvertes.fr/hal-00578694 2012.
