# Supplementary material for: Goodness-of-Fit Tests and Nonparametric Adaptive Estimation for Spike Train Analysis
Source: J Math Neurosci. 2014 Apr 17;4:3. doi: 10.1186/2190-8567-4-3 (PMC4021619; doi:10.1186/2190-8567-4-3)
Supplement: Additional file 3 — Adaptive properties of the Lasso estimate for Hawkes processes: the oracle inequality satisfied by the Lasso estimate fˆB (see Sect. 3.3.3) (PDF 84.5 KB) [file 2190-8567-4-3-S3.pdf]

Additional File 3 of "Goodness-of-fit tests and nonparametric adaptive estimation for spike train analysis": adaptive properties of the Lasso estimate for Hawkes processes

Patricia Reynaud-Bouret*[1] and Vincent Rivoirard[2] and Franck Grammont[1] and Christine Tuleau-Malot[1]

[1]Univ. Nice Sophia Antipolis, CNRS, LJAD, UMR 7351, 06100 Nice, France
[2]CEREMADE UMR CNRS 7534, Université Paris Dauphine, Place du Maréchal De Lattre De Tassigny, 75775 PARIS Cedex 16, France

Email: Patricia Reynaud-Bouret*- reynaudb@unice.fr; Vincent Rivoirard - rivoirard@ceremade.dauphine.fr; Franck Grammont - grammont@unice.fr; Christine Tuleau-Malot - malot@unice.fr;

*Corresponding author

Theorem 1 of [1] does not explicit particular basis on which the interaction functions are expansed. It is stated in its general form as follows. Note that [1] is a proceedings and therefore no proof has been given of the result in [1]. If n i.i.d. trials are recorded, each trial i corresponds to the observation of $N_i = (N_i^{(1)}, ..., N_i^{(M)})$, the multivariate Hawkes process whose intensity is given by the predictable transformation denoted ψ_i. Furthermore, to each trial i, we can associate an intensity λ_i and a contrast $\gamma^{(i)}$. The global least-squares contrast over the n trials can also be seen as

$$\gamma_n(f) = \sum_{i=1}^{n} \gamma^{(i)}(f). \tag{1}$$

We use the following notation: for any predictable processes $H = (H_i^{(1)}, ..., H_i^{(M)})_{i=1,...n}$, $K = (K_i^{(1)}, ..., K_i^{(M)})_{i=1,...,n}$, set

$$H \bullet N = \sum_{i=1}^{n} \sum_{m=1}^{M} \int_{T_1}^{T_2} H_{i,t}^{(m)} dN_i^{(m)}(t), \tag{2}$$

$$H \diamond K = \sum_{i=1}^{n} \sum_{m=1}^{M} \int_{T_1}^{T_2} H_{i,t}^{(m)} K_{i,t}^{(m)} dt, \tag{3}$$

and $H^{\diamond 2} = H \diamond H$.

In general, we use a dictionary Φ of known functions of $\mathcal{H}$ and we only consider linear combinations of functions of Φ for estimating f^*:

$$f_a = \sum_{\varphi \in \Phi} a_\varphi \varphi, \quad \text{for} \quad a \in \mathbb{R}^\Phi. \tag{4}$$

Then, by linearity of ψ, one can rewrite (1) as

$$\gamma_n(f_a) = -2a'b_n + a'G_n a, \tag{5}$$

where for any φ and $\tilde{\varphi}$ in Φ,

$$(b_n)_\varphi = \psi(\varphi) \bullet N \quad \text{and} \quad (G_n)_{\varphi,\tilde{\varphi}} = \psi(\varphi) \diamond \psi(\tilde{\varphi}).$$

Given a vector of positive weights d, the *Lasso estimate* of f^* is $\tilde{f}_n := f_{\tilde{a}_n}$ where $\tilde{a}_n$ is a minimizer of the following ℓ_1-penalized least-square contrast:

$$\tilde{a}_n \in \arg\min_{a \in \mathbb{R}^\Phi} \{-2a'b_n + a'G_n a + 2d'|a|\}. \tag{6}$$

Then Theorem 1 of [1] is stated as follows:

Theorem 1. *We introduce the following two events:*

$$\Omega_{V,B} = \{\forall\, \varphi \in \Phi, \sup_{t \in [T_1,T_2], m, i} |\psi_{i,t}^{(m)}(\varphi)| \leq B_\varphi \text{ and } (\psi(\varphi))^2 \bullet N \leq V_\varphi\},$$

for positive deterministic constants B_φ and V_φ and

$$\Omega_c = \left\{\forall\, a \in \mathbb{R}^\Phi, \quad a'G_n a \geq c\, a'a\right\}, \tag{7}$$

for a positive constant c. Let x and ε be strictly positive constants and for all $\varphi \in \Phi$,

$$d_\varphi = \sqrt{2(1+\varepsilon)\hat{V}_\varphi^\mu x} + \frac{B_\varphi x}{3}, \tag{8}$$

with

$$\hat{V}_\varphi^\mu = \frac{\mu}{\mu - \phi(\mu)}\ (\psi(\varphi))^2 \bullet N + \frac{B_\varphi^2 x}{\mu - \phi(\mu)}$$

for a real number μ such that $\mu > \phi(\mu)$, where $\phi(\mu) = \exp(\mu) - \mu - 1$. Then, with probability larger than

$$1 - 4\sum_{\varphi \in \Phi} \left(\frac{\log\left(1 + \frac{\mu V_\varphi}{B_\varphi^2 x}\right)}{\log(1+\varepsilon)} + 1\right) e^{-x} - \mathbb{P}((\Omega_{V,B} \cup \Omega_c)^c),$$

the following inequality holds

$$[\psi(\tilde{f}_n) - \lambda]^{\diamond 2} \leq C \inf_{a \in \mathbb{R}^\Phi} \left\{[\psi(f_a) - \lambda]^{\diamond 2} + \frac{1}{c}\sum_{\varphi \in S(a)} d_\varphi^2\right\},$$

where C is an absolute positive constant and where $S(a)$ is the support of a, i.e. its coordinates with non-zero coefficients.

Proof. We use the notation of [2] and transposition of this notation. First by scaling the data, it is always possible to assume that $A = 1$. We have at hand $n \times M$ point processes $N_m^{(i)}$. In the more general case, we need to model each $\lambda^{(m,i)}$, intensity of $N_m^{(i)}$, by a

$$\psi_{f_n}^{(m,i)}(t) = \mu^{(m,i)} + \sum_{\ell,j} \int_{-\infty}^{t-} g_{\ell,j}^{(m,i)}(t-u)dN_j^{(\ell)}(u),$$

where f_n belongs to $\mathcal{H}_n$ which replaces the space $\mathcal{H}$:

$$\mathcal{H}_n = (\mathbb{R} \times \mathbb{L}_2((0,1])^{nM})^{nM} = \Big\{ f_n = \Big((\mu^{(m,i)}, (g_{\ell,j}^{(m,i)})_{\ell=1,\dots,M,\ j=1,\dots,n} \big)_{m=1,\dots,M,\ i=1,\dots n} \Big) : \\ g_{\ell,j}^{(m,i)} \text{ with support in } (0,1] \text{ and } \|f_n\|^2 = \sum_{m,i} (\mu^{(m,i)})^2 + \sum_{m,i} \sum_{\ell,j} \int_0^1 g_{\ell,j}^{(m,i)}(t)^2 \mathrm{d}t < \infty \Big\}.$$

For every $f_n = \Big((\mu^{(m,i)}, (g_{\ell,j}^{(m,i)})_{\ell=1,\dots,M,\ j=1,\dots,n})_{m=1,\dots,M,\ i=1,\dots n} \Big)$ in $\mathcal{H}_n$, we denote for each m and i,

$$f_n^{(m,i)} = (\mu^{(m,i)}, (g_{\ell,j}^{(m,i)})_{\ell=1,\dots,M,\ j=1,\dots,n}.$$

In the same way for every $f = \Big((\mu^{(m)}, (g_{\ell,j}^{(m)})_{\ell=1,\dots,M,\ j=1,\dots,n} \Big)_{m=1,\dots,M}$ in $\mathcal{H}$,we denote for each m and i,

$$f^{(m)} = (\mu^{(m)}, (g_{\ell,j}^{(m)})_{\ell=1,\dots,M,\ j=1,\dots,n}.$$

Now our dictionary Φ of $\mathcal{H}$ can be transformed into a dictionary Φ_n of $\mathcal{H}_n$ by stating that for any φ in Φ we associate a φ_n in $\mathcal{H}_n$ such that for all i, m, $\varphi_n^{(m,i)} = \varphi^{(m)}$. Therefore it is easy to see that the vector b of [2] associated to f_n is actually our vector b_n and that the matrix G of [2] is our matrix G_n. The V and B are in the same way translated and the present result is a pure application of Theorem 2 of [2] □

References

1. Reynaud-Bouret P, Rivoirard V, Tuleau-Malot C: **Inference of functional connectivity in Neurosciences via Hawkes processes**. In *1st IEEE Global Conference on Signal and Information Processing* 2013, Austin Texas.
2. Hansen N, Reynaud-Bouret P, Rivoirard V: **Lasso and probabilistic inequalities for multivariate point processes**. *to appear in Bernoulli* 2013. [Http://arxiv.org/abs/1208.0570].
